# Automated extraction of speech and turn-taking parameters in autism allows for diagnostic classification using a multivariable prediction model

**DOI:** 10.3389/fpsyt.2023.1257569

**Published:** 2023-11-06

**Authors:** I. S. Plank, J. C. Koehler, A. M. Nelson, N. Koutsouleris, C. M. Falter-Wagner

**Affiliations:** ^1^Department of Psychiatry and Psychotherapy, LMU University Hospital, LMU Munich, Munich, Germany; ^2^Max Planck Institute of Psychiatry, Munich, Germany; ^3^Institute of Psychiatry, Psychology and Neuroscience, King’s College, London, United Kingdom

**Keywords:** speech, turn-taking, diagnostic classification, autism, prediction model, conversation

## Abstract

Autism spectrum disorder (ASD) is diagnosed on the basis of speech and communication differences, amongst other symptoms. Since conversations are essential for building connections with others, it is important to understand the exact nature of differences between autistic and non-autistic verbal behaviour and evaluate the potential of these differences for diagnostics. In this study, we recorded dyadic conversations and used automated extraction of speech and interactional turn-taking features of 54 non-autistic and 26 autistic participants. The extracted speech and turn-taking parameters showed high potential as a diagnostic marker. A linear support vector machine was able to predict the dyad type with 76.2% balanced accuracy (sensitivity: 73.8%, specificity: 78.6%), suggesting that digitally assisted diagnostics could significantly enhance the current clinical diagnostic process due to their objectivity and scalability. In group comparisons on the individual and dyadic level, we found that autistic interaction partners talked slower and in a more monotonous manner than non-autistic interaction partners and that mixed dyads consisting of an autistic and a non-autistic participant had increased periods of silence, and the intensity, i.e. loudness, of their speech was more synchronous.

## 1. Introduction

Speech as a form of communication is unique to humans. According to Ferdinand de Saussure, it is based on signs combining acoustic forms (the signifier) with meaning (the signified) (1). All signifiers can vary in their production to add contextual meaning to their signified. Important speech features like pitch, referring to the tone of speech, intensity, referring to the volume of speech, and articulation rate, referring to the speed of speech, are all influenced by the affective and mental state of the speaker (2, 3). Therefore, they strongly influence how a certain utterance is perceived: Meaning is not only *what* we say, but *how* we say it.

Autism spectrum disorder (ASD) is a neurodevelopmental disorder that entails symptoms regarding communication, social behaviour and behavioural rigidity (4). Speech can be completely absent in autistic people. Even in verbal individuals, speech of autistic people differs to that of non-autistic people (5, 6). One of the most common diagnostic instruments for ASD, the Autism Diagnostic Observation Schedule [ADOS(R); (7)], highlights that both changes in prosody and speech rate may indicate ASD, amongst other verbal behaviours.

A recent meta-analysis evaluated the alterations of speech features in ASD (5). The authors found that pitch differs between autistic and non-autistic people in terms of increased mean and variance. However, results concerning intensity and speech rate were more equivocal. For both domains, many included studies did not show differences between autistic and non-autistic people, while other studies found effects, though not all of them in the same direction. The meta-analysis did not include studies that investigated variance of intensity over the course of a conversation. In another systematic review (8), two studies investigating variance of intensity were mentioned, one of which did not find differences in intensity range (9), and the other found decreased standard deviation of intensity (10). It is important to note that both Fusaroli et al. (8) and Asghari et al. (5) included various modes of speech production, ranging from spontaneous production over narration to social interactions. Additionally, both included all age ranges, so it is possible that not all outcomes apply to adults.

In addition to the importance of speech differences, autistic people report having difficulties with small talk and are perceived as more awkward in conversations (11–14). Since small talk and conversations with strangers are essential for building connections with others, it is important to understand how autistic verbal behaviours differ from non-autistic verbal behaviours in these situations. Reciprocal communication is characterised by a to and fro of speaking and listening. Successful turn-taking not only requires mutual prediction of an upcoming transition point but also a minute concertation of behaviours between interaction partners allowing them to be in sync (15). The length of turn-taking gaps can be an estimate of how in sync interaction partners were and is associated with social connection (16). If two strangers lose their flow, they tend to feel awkward and try to fill the silence (17). A recent study by Ochi et al. (18) found increased turn-taking gaps and more silence vs. talking as measured by the silence-to-turn ratio (19). However, the sample consisted of only male autistic and non-autistic participants, and it is unclear whether the results generalise to people of other genders. Therefore, it is especially important to investigate turn structure in a more general sample to assess the quality of verbal communication.

Finally, the investigation of speech features should be extended to include the temporal fine-tuning within interaction dyads, given the increasing literature showing reduced interactional synchrony in dyads of one autistic and one non-autistic compared to two non-autistic interaction partners [e.g. (20); for a review, see (21)]. Behavioural synchrony is the product of coordination between interaction partners. This coordination can be achieved by the interaction partners adapting their behaviour to each other. Synchrony of speech features is well documented (22–25); however, research investigating speech synchrony in autistic people is scarce. Ochi et al. (18) found that non-autistic participants showed more synchrony between the ADOS interviewer’s intensity and their own than autistic participants, but they found no differences regarding synchrony of pitch. Wynn et al. (26) altered the speed in trial prompts and found that non-autistic adults adapted the speed of their answer in the corresponding trial, while autistic adults and children did not. Both studies show that interpersonal coordination of speech features is a promising avenue to investigate differences in verbal interaction between autistic and non-autistic people.

Additionally, a recent study also used parts of ADOS interviews to investigate classification between autistic and non-autistic children based on synchrony of speech features (27). They extracted lexical features and calculated the similarity of the lexical content of the interviews. Machine learning classifiers were able to predict whether a child was diagnosed with ASD with better accuracy when the synchrony measures were added to the model as compared to a model that only included individual speech features. However, in that study, the ADOS was used both for creating the true labels and to extract features for the classification, risking circularity that might artificially inflate accuracies. Therefore, it is vital to assess the performance of classifiers with features extracted from data that is independent from the diagnostic process. In a recent study using automatically extracted interpersonal synchrony of motion quantity and facial expressions, we show that pursuing more naturalistic study designs can yield high classification accuracy of almost 80% (28). If these results can be extended to speech and interactional features of verbal communication in adults, this would provide a low-tech and scalable route to assist clinicians with the diagnosis of ASD.

This study design fills the outlined gaps in the literature by extracting speech parameters with an automated pipeline from naturalistic conversations that are independent of the diagnostic assessment to avoid any circularity in the classification procedure. The automated extraction of features increases objectivity, specificity and applicability of the pipeline to a variety of conversational paradigms. The main aim of the current study was (i) to determine the potential of speech coordination as a diagnostic marker for ASD. Additionally, we defined two secondary aims: (ii) to describe individual speech feature differences, and (iii) interactional speech differences that can help explain the classification power. Concerning our main aim (i), we expected that a multivariable prediction model would be able to classify dyad type based on individual speech and dyadic conversational features, thereby offering an exciting possibility for assisting diagnostics of ASD. On the individual level regarding our aim (ii), we expected that autistic and non-autistic individuals would differ in their pitch variance, intensity variance and articulation rate. Additionally, we computed turn-based adaptation of pitch, intensity and articulation rate and expected increased turn-based adaptation in non-autistic compared to autistic individuals. On the dyadic level regarding our aim (iii), we hypothesised that interactional differences would be found in silence-to-turn ratios, turn-taking gaps as well as time-course synchrony of pitch and intensity.

## 2. Materials and methods

This study is part of a larger project to find diagnostic markers for ASD. The preregistration of the hypotheses regarding aim (ii) and (iii) can be retrieved from OSF.^1^ Preprocessing was performed using Praat *6.2.09* (29), the uhm-o-meter scripts provided by De Jong et al. (30, 31) and R *4.2.2* (32) in Rstudio *2022.12.0* (33). The Bayesian analysis was performed in R and JASP *0.16.4* (34). The machine learning analysis was conducted with the NeuroMiner toolbox *1.1* (35) implemented in MATLAB *R2022b* (36) and Python *3.9*.^2^ All code used to preprocess and analyse the data can be found on GitHub.^3^ We report our prediction model following the Transparent Reporting of a Multivariable Prediction Model for Individual Prognosis or Diagnosis (TRIPOD) guidelines (37).

### 2.1. Participants

**Figure 1:**
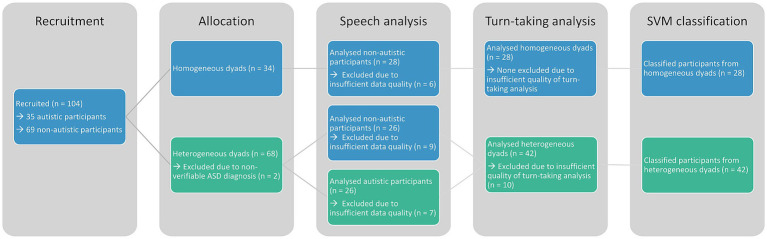
This consort chart shows the recruitment and exclusion of participants. All sample sizes are given per participant and not per dyad. Colours indicate the group affiliation at the respective analysis step.

We recruited 35 autistic and 69 non-autistic participants from the general population and the outpatient clinic at the LMU University Hospital Munich by posting flyers at the university and at the hospital as well as distributing them online on social media and mailing lists. Of these participants, 26 autistic (*mean* age = 34.85 ± 12.01 years, 17 male) and 54 non-autistic (*mean* age = 30.80 ± 10.42 years, 21 male) participants were analysed (Figure 1). Non-autistic participants were recruited to match the overall gender and age distribution of the autistic sample. This sample is a subset of the sample analysed by Koehler et al. (28) containing all participants with sufficient audio data quality.

All participants were between 18 and 60 years old, had no current neurological disorder and had an IQ above 70 based on verbal and non-verbal IQ tests (38, 39). For each autistic participant, an ASD diagnosis (F84.0 or F84.5) according to the ICD-10 (40) was confirmed by evaluating the diagnostic report. All non-autistic participants had no current or previous psychiatric diagnosis and no intake of psychotropic medication. Autistic and non-autistic participants did not differ credibly in age, verbal [measured with the Mehrfachwahl-Wortschatz-Intelligenztest, MWT-B; (38)] or nonverbal IQ [measured with the Culture Fair Intelligence Test, CFT-20-R; (39)], but they differed credibly on the Adult Dyspraxia Checklist [ADC; (41)], the Autism Quotient [AQ; (42)], the Beck’s Depression Inventory [BDI; (43)], Self-Monitoring Scale [SMS-short; (44)], the Saarbrückener Persönlichkeitsfragebogen [SPF; (45), German version of the Interpersonal Reacitivity Index, IRI, (46)] and the Toronto Alexithymia Scale [TAS-20; (47); see Table 1]. Two autistic participants had a comorbid diagnosis of attention deficit hyperactivity disorder (ADHD), nine of an affective disorder and five of a neurotic stress-related or somatoform disorder. The study was conducted in accordance with the Declaration of Helsinki and approved by the ethics committee of the medical faculty of the LMU. All participants provided written, informed consent and received a monetary compensation for their participation.

**Table 1:** Mean and standard deviation of the autistic and non-autistic samples analysed in this study as well as group comparisons performed with Bayesian Mann–Whitney U tests based on 10,000 samples.

	Autistic	Non-autistic	Log(BF_10_)	W
Age	34.85 ± 12.01	30.80 ± 10.42	−1.028	564.50
IQ – nonverbal	115.35 ± 22.96	117.07 ± 15.21	−1.384	716.50
IQ – verbal	112.12 ± 15.01	113.96 ± 16.53	−1.297	743.00
ADC	50.12 ± 16.06	15.57 ± 8.99	9.339	61.50
AQ	33.00 ± 8.41	14.26 ± 4.55	8.863	65.50
BDI	18.35 ± 12.36	3.94 ± 3.96	8.137	139.00
SMS-short	5.92 ± 2.86	9.54 ± 2.98	5.080	1,136.00
SPF	36.77 ± 6.62	45.43 ± 5.35	6.498	1,214.00
TAS-20	61.27 ± 11.74	36.91 ± 7.61	8.359	77.00

Note. ADC, Adult Dyspraxia Scale; AQ, Autism Quotient; BDI, Beck’s Depression Inventory; SMS-short, Self-Monitoring Scale; SPF, Saarbrückener Persönlichkeitsfragebogen; TAS-20, Toronto Alexithymia Scale.

### 2.2. Experimental procedure

After giving informed consent, blood samples were taken, followed by demographics and the intelligence assessments. Throughout the session, participants completed the above listed questionnaires. They also performed a task assessing emotion recognition [BERT, (48)]. In addition, some of the participants took part in a separate study measuring endocrinology and effects of social ostracism.

We paired participants in either mixed dyads consisting of one autistic and one non-autistic participant or non-autistic dyads. Participants were paired based on availability regardless of age and gender. Dyads did not differ in average age or age difference between the interaction partners. However, there was strong evidence in favour of a difference in gender composition (mixed dyads: *mean* age = 33.15 ± 7.72, *mean* age difference = 12.69 ± 9.18 [1 to 32 years], 15% female, 35% male and 50% gender-mixed dyads; non-autistic dyads: *mean* age = 30.18 ± 8.22, *mean* age difference = 10.64 ± 11.15 [1 to 31 years], 50% female and 50% gender-mixed; for statistical values see Supplementary material S1.1). We did not disclose their interaction partner’s diagnostic status to them. The dyads engaged in two 10-minute long conversations: one about their hobbies and one fun task in which they were asked to plan a menu consisting of food and drinks that they both disliked (49). On the one hand, we chose the *hobbies* task because special interests are a core symptom of ASD (4). On the other hand, the *meal planning* task facilitates a more collaborative interaction and has been shown to promote increased synchrony in non-autistic dyads (20). The experimenter left the room during the conversations. After both conversation tasks, participants were asked to rate the quality of their interactions. During the COVID-19 pandemic, testing had to be moved to a different room after nine dyads and a plexiglass was placed between the participants as a health and safety measure. Participants did not wear masks during the conversations and the quality of interactions was rated equal before and after the measures had been put into place (28).

We captured participants’ behaviour via multiple channels. The current study focuses on speech coordination captured with one recording device to which two separate microphones were connected (t.Bone earmic 500 with ZoomH4n recorder). The nonverbal communication parameters, body movement captured by a scene camera (Logitech C922), facial expressions captured by two face cameras (Logitech C922), heart rate and electrodermal activity captured by wearables (Empatica E4), as well as the analysis of the blood samples were outside of the scope of the current analysis and published elsewhere (28). For more details on the data collection procedure, please consult Supplementary material S1.2.

### 2.3. Preprocessing

**Figure 2:**
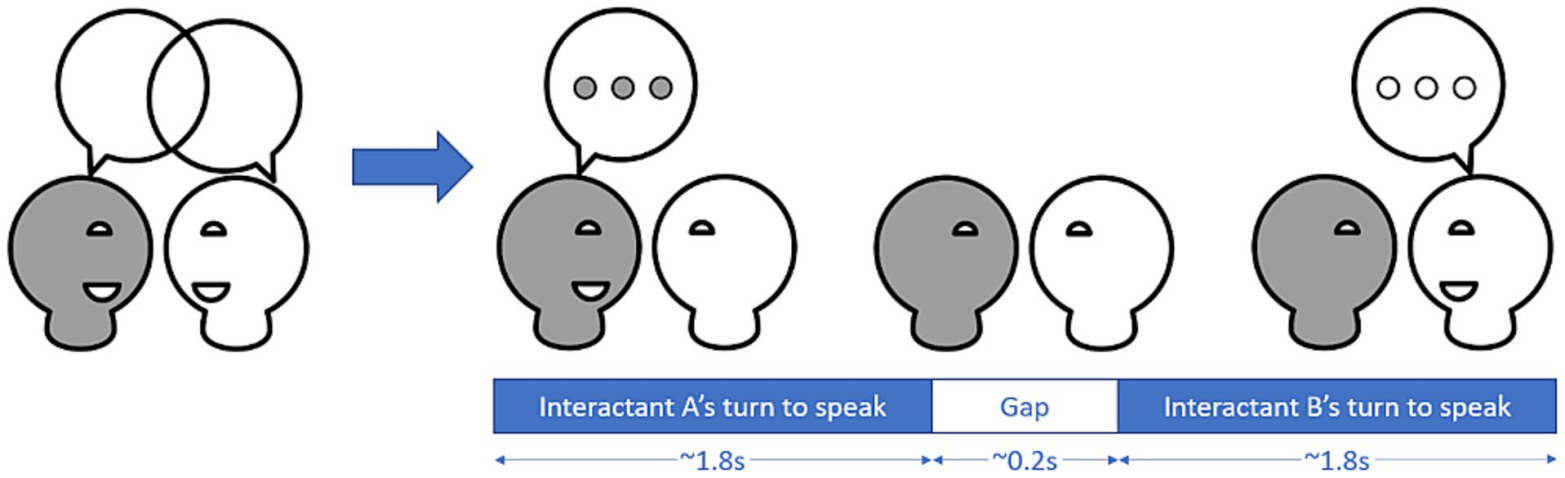
Conversations can be broken down into turns where one of the interactant is speaking and gaps between the turns. In a large-scale study, Templeton et al. (16) found that turns in an unstructured conversation between strangers have a median length of 1.8 s, while the median length of gaps was about 0.2 s.

We extracted individual phonetic features for each task and participant using praat (29) (for more details, see Supplementary material S1.3). We calculated pitch and intensity synchrony with rMEA’s cross-correlation function to calculate windowed cross-lagged correlations (WCLC) using the same window length of 16 s, step size of 8 s and lag of 2 s as Ochi et al. (18). We used the uhm-o-meter (30, 31) to extract turns from conversations, with a turn defined as all speaking instances of one interactant until the end of the speaking instance preceding the next speaking instance of someone else (see Figure 2). For each turn, we calculated turn-taking gap, average pitch, average intensity and number of syllables to calculate articulation rate. Additionally, we used turn-based information to calculate how much each participant adapted their pitch, intensity and articulation rate to the pitch, intensity and articulation rate of the previous turn.

### 2.4. Comparison of synchrony with pseudosynchrony

We used segment shuffling as described by Moulder et al. (50) to determine whether synchrony and turn-based adaptation calculations are credibly different from their corresponding pseudo values (see also Supplementary material S3). These pseudo values are created by randomly shuffling one of the interactant’s data and then computing synchrony between the shuffled and real data. For each synchrony and turn-based adaptation value, we computed the average of 100 pseudosynchrony or pseudoadaptation values. Then, we used a Bayesian paired t-test as implemented in the BayesFactor package to compare the values. There was evidence in favour of the hypotheses that pitch and intensity synchrony as well as turn-based adaptation of pitch, intensity and articulation rate were all credibly higher than the corresponding pseudo values (see Table 2). This indicates that the obtained synchrony values exceeded chance coordination.

**Table 2:** Comparison of synchrony and turn-based adaptation values with their corresponding pseudo values.

	Mean and SD of values	Mean and SD of pseudo values	Log(BF_10_)
Individual adaptation			
Turn-based pitch	0.121 ± 0.107	0.087 ± 0.020	4.561
Turn-based intensity	0.146 ± 0.086	0.091 ± 0.017	20.977
Turn-based articulation rate	0.138 ± 0.096	0.099 ± 0.019	7.017
Dyadic synchrony			
Pitch	0.197 ± 0.022	0.190 ± 0.003	1.423
Intensity	0.368 ± 0.048	0.164 ± 0.010	104.491

### 2.5. Support vector machine for classification

We used a linear L2-regularised L2-loss support vector machine (SVM) as implemented by LIBLINEAR in NeuroMiner to predict each individual’s participation in either a non-autistic or mixed dyad to address our main aim (i). SVMs have not only been applied to classify several psychiatric diagnoses (51, 52), but have been specifically applied to predict ASD based on interactional data (28, 53, 54). Therefore, we chose to use an SVM to allow for comparability with previous results and decided on a linear SVM for its computational speed (55). We combined the SVM with a L2 or ridge regression since this seems to perform better with correlated predictors (56). The SVM algorithm optimises a linear hyperplane to achieve maximum separability between non-autistic and mixed dyads in the high-dimensional feature space. Separability was assessed using balanced accuracy, which equally weighs sensitivity (ratio of true positives to the sum of the true positives and false negatives) and specificity (ratio of true negatives to the sum of the true negatives and false positives). We used hyperplane weighting to account for unbalanced sample sizes where the misclassification penalty for the smaller sample is increased (35). The algorithm optimises the hyperplane so that the geometric margin between most similar instances of opposite classes (i.e. the support vectors) is maximised, thus increasing generalisability to new observations following the principles of statistical learning theory (57–59). The SVM algorithm determines dyad membership by the position of an individual with respect to the optimally separating hyperplane (OSH), while the individual’s decision score measures the geometric distance to the OSH with higher absolute values indicating a more pronounced expression of the given separating pattern. This decision score then determines the label assigned to an individual, specifically if the individual was part of a non-autistic or mixed dyad. All features (see Table 3) were scaled from −1 to 1 and pruned to exclude zero variance features as recommended by the NeuroMiner manual. We used a repeated, nested stratified cross-validation (CV) structure to account for the unbalanced sample sizes and the dyadic nature of the data, ensuring that two interactants of the same dyad were always in the same fold and that the ratio of interactants from mixed and non-autistic dyads was consistent in all folds. The CV structure consisted of two loops with the outer loop being implemented in seven folds and 10 permutations and the inner loop with 10-fold and one permutation. The outer loop iteratively held back five dyads to validate the algorithm on unseen data, while the rest of the dyads was included in the inner loop. Here, three dyads were held back for validation. The decision scores of the test data of the inner loop were additionally post-hoc optimised according to the receiver operator function. Last, we used label permutation testing while keeping the cross-validation structure intact to assess whether the resulting SVM performance was above chance (5,000 permutations, *α_Bonferroni-corrected_* = 0.007).

**Table 3:** List of individual and dyadic features.

Individual	Dyadic
Articulation rate	Number of turns
Number of pauses	Silence-to-turn ratio
Number of syllables	Speech rate
Phonation time	Synchrony of intensity
Turn-based adaptation of articulation rate	Synchrony of pitch
Turn-based adaptation of intensity	Turn-taking gap
Turn-based adaptation of pitch	
Variance of intensity	
Variance of pitch	

Note. All features were entered for the meal planning and the hobbies task separately, resulting in 30 features. Articulation rate refers to the number of syllables per phonation time, while speech rate refers to the number of syllables per total time (phonation time and silence).

### 2.6. Bayesian analysis

We tested our hypotheses regarding aims (ii) and (iii) using Bayesian repeated-measures ANOVAs as implemented in JASP. Each ANOVA included one within-subjects factor (task: *meal planning*, *hobbies*) and one between-subjects factor, either diagnostic status (autistic, non-autistic) or dyad type (mixed, non-autistic). We checked for equality of variance and visually inspected whether the residuals were normally distributed. In the case of violations of these assumptions, we computed a non-parametric alternative and compared the results. We used the Bayes Factor to assess the strength of evidence for or against a model or inclusion of a factor. The Bayes Factor is the ratio of marginal likelihoods, thereby quantifying how much more or less likely one model is than the other. We interpreted the logarithmic Bayes Factor according to Jeffrey’s scheme (60). For example, if a model is more than 100 times as likely [*Log(BF)* > Log(100) = 4.6], we consider this *decisive* evidence in favour of this model [very strong: *Log(BF)* > 3.4; strong: *Log(BF)* > 2.3; moderate: *Log(BF)* > 1.1; anecdotal: *Log(BF)* > 0]. We use the logarithm of the Bayes Factor because it leads to symmetric thresholds: a *Log(BF)* of 4 signifies very strong evidence in favour of a model and a *Log(BF)* of −4 the same strength of evidence against a model.

There was a credible difference between the gender composition of the non-autistic and the mixed dyads due to no non-autistic male dyads. Since studies have shown differences between genders with regard to language in ASD (61–63), we repeated all group comparisons on the dyad level excluding the male mixed dyads to ensure that possible differences are not driven by gender composition.

## 3. Results

### 3.1. Performance of support vector machine for classification

**Figure 3:**
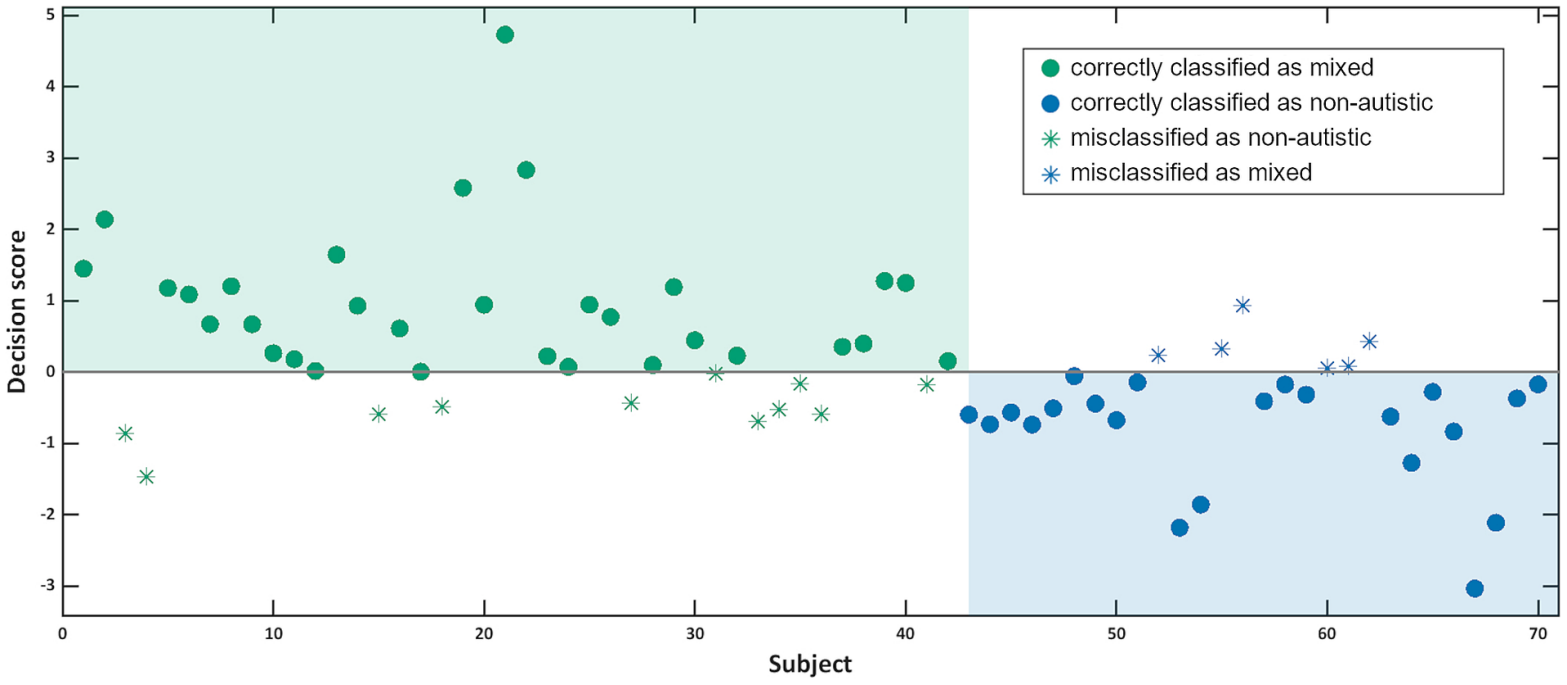
This graph shows for each participant the decision score calculated by the SVM classifier with participants from non-autistic dyads in blue and participants from mixed dyads in green. Filled circles were correctly categorised by the classifier, while empty circles were misclassified.

Our SVM algorithm was able to distinguish between individuals from a non-autistic and a mixed dyad with 76.2% balanced accuracy on the basis of both individual and dyadic speech and communication features. Specifically, 78.6% of the individuals from a non-autistic dyad were correctly labelled as such (specificity), while 73.8% of the individuals from a mixed dyad were assigned the correct label (sensitivity, see Figure 3). While this model performs significantly above chance levels (*p* < 0.001; area under the curve: 0.81 [CI 0.72–0.92]; please consult Supplementary material S2 for more details on the SVM classifier), it does not outperform a model trained on synchrony of facial expressions with a balanced accuracy of 79.5% or a stacked model with a balanced accuracy of 77.9% including multiple movement parameters automatically extracted from video recordings of dyadic interactions (28).

### 3.2. Group comparisons on the individual and the dyad level

#### 3.2.1. Individual differences between autistic and non-autistic participants

Autistic participants differed from non-autistic participants in their speech features as evidenced by the results of the Bayesian ANOVAs.

##### 3.2.1.1. Pitch

Pitch variance was best explained by a model including task and diagnostic status but not the interaction of the two [*Log(BF_10_)* = 5.612]. The analysis of effects across matched models revealed very strong evidence for the inclusion of task and anecdotal evidence for the inclusion of diagnostic status [task: *Log(BF_incl_)* = 4.455; diagnostic status: *Log(BF_incl_)* = 1.030]. There was anecdotal evidence against the inclusion of the interaction [task × diagnostic status: *Log(BF_incl_)* = −0.510]. However, the Q-Q plot of the residuals revealed deviations from the normal distribution and the variances were not homogeneous. Therefore, we computed a Bayesian Mann–Whitney U test to determine whether the anecdotal evidence in favour of an effect of diagnostic status can be reproduced with a non-parametric test, which was the case [*Log(BF_10_)* = 0.888, *W* = 439.00]. Pitch variance was increased in non-autistic compared to autistic participants.

##### 3.2.1.2. Intensity

**Figure 4:**
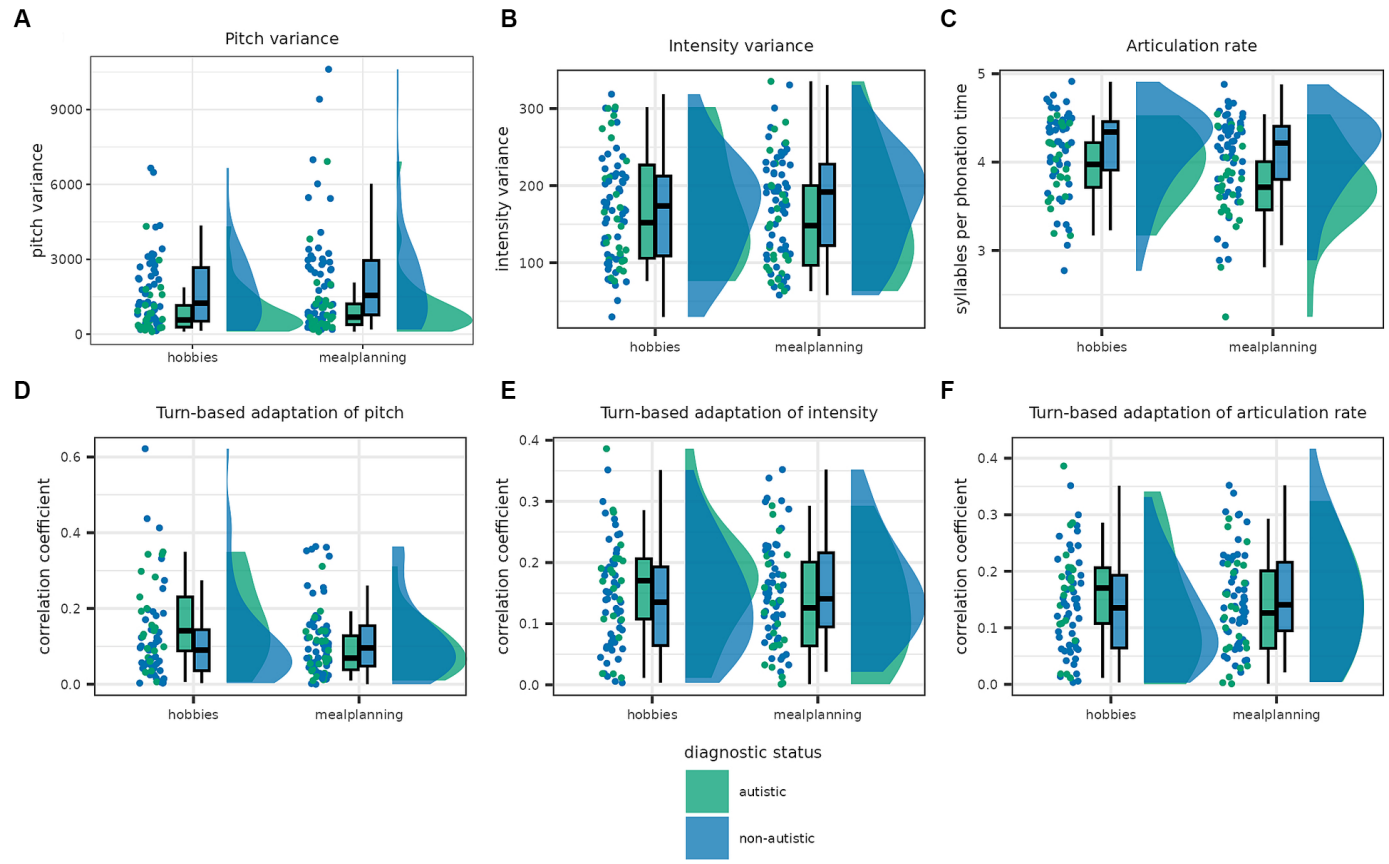
This graph shows the distribution of individual features in the autistic and non-autistic participants as scatterplots, density plots and box plots. The boxes show the interquartile range and the median, while the whiskers show 1.5 times the interquartile range added to the third and subtracted from the first quartile. In the first row, panel **(A)** shows pitch variance, panel **(B)** intensity variance and the panel **(C)** articulation rate. All three features were increased in non-autistic compared to autistic participants. The second row shows the amount participants adapted their pitch **(D)**, intensity **(E)** and articulation rate **(F)** to the previous turn. There were no significant differences in adaption of all three speech factors between autistic and non-autistic participants.

The best model describing intensity variance was the full model including the predictors task and diagnostic status as well as their interaction [*Log(BF_10_)* = 3.205]. The analysis of effects across matched models revealed that this was mainly driven by the interaction with decisive evidence in favour of the interaction effect and moderate and anecdotal evidence against task and diagnostic status, respectively [task × diagnostic status: *Log(BF_incl_)* = 5.163; task: *Log(BF_incl_)* = −1.544; diagnostic status: *Log(BF_incl_)* = −0.436]. Specifically, while intensity variance of autistic participants was increased in the *hobbies* condition, the reverse was true for non-autistic participants (see Figure 4).

##### 3.2.1.3. Articulation rate

Articulation rate was again best described by the full model including task, diagnostic status and the interaction [*Log(BF_10_)* = 6.727], with moderate evidence in favour of including diagnostic status as well as strong evidence in favour of including task and the interaction [task × diagnostic status: *Log(BF_incl_)* = 2.517; task: *Log(BF_incl_)* = 2.656; diagnostic status: *Log(BF_incl_)* = 1.517]. Articulation rate was faster in non-autistic than autistic participants.

##### 3.2.1.4. Turn-based adaptation

Last, the null model outperformed all alternative models with anecdotal evidence in favour of the null model for turn-based adaptation of pitch and intensity (see Supplementary material S4). In the case of adaptation of articulation rate, there was anecdotal evidence in favour of the model including task but no other predictor [*Log(BF_10_)* = 1.072] with higher articulation rate in the *meal planning* condition. Since the residuals were not normally distributed, we performed non-parametric tests which confirmed no effect of diagnostic status on all three adaptation parameters (see Supplementary material S4).

#### 3.2.2. Dyadic differences between non-autistic and mixed dyads

**Figure 5:**
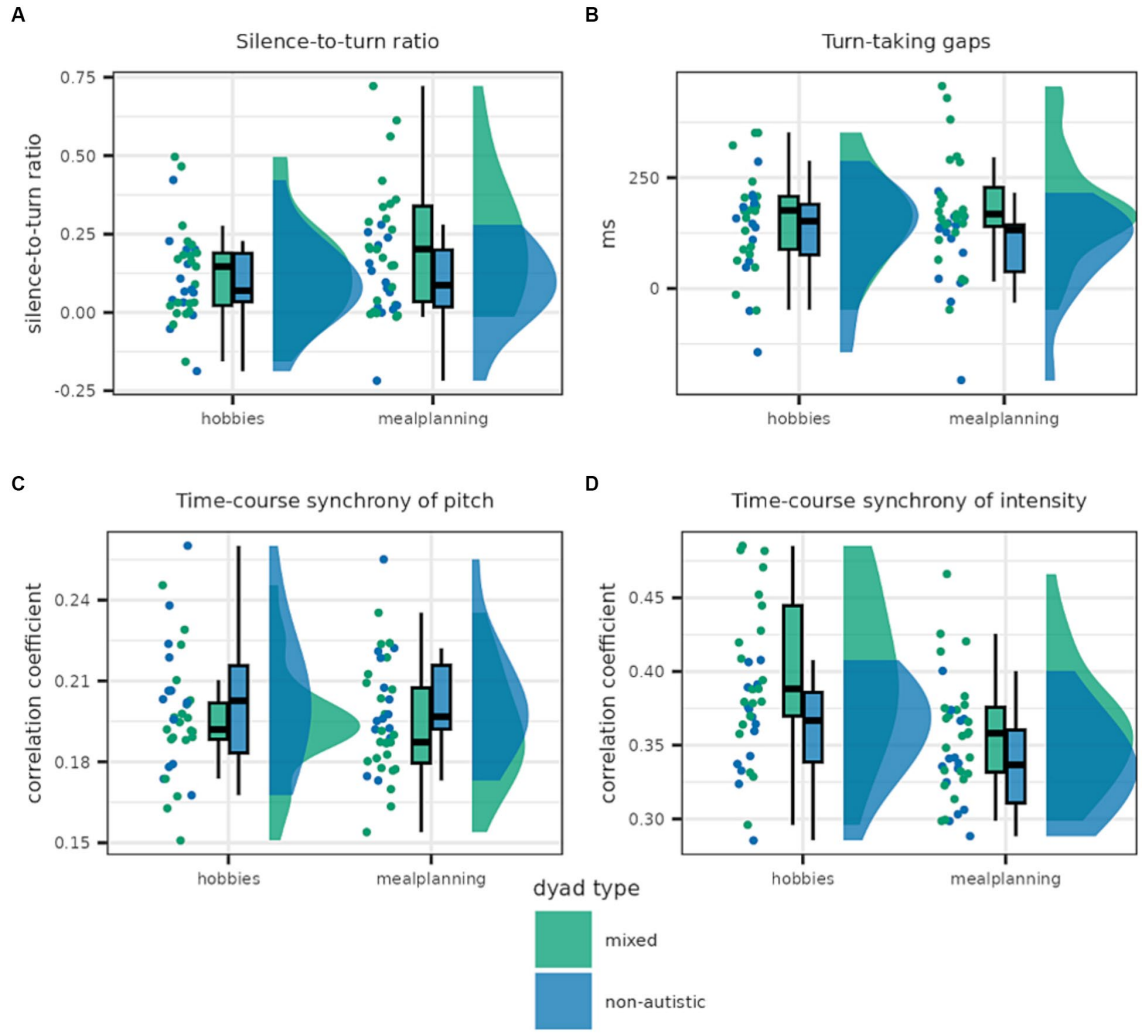
This graph shows the distribution of dyadic features for mixed and non-autistic dyads. Panel **(A)** shows the silence-to-turn ratio which was higher in mixed compared to non-autistic dyads. Panel **(B)** shows turn-taking gaps which were, on average, longer in mixed dyads. The lower panels show time-course synchrony of pitch **(C)** and intensity **(D)** with the latter being higher in mixed dyads.

Some interactional features differed between non-autistic and mixed dyads; however, others were comparable in both dyad types (see Figure 5).

##### 3.2.2.1. Silence-to-turn ratio

The silence-to-turn ratio was best predicted by the full model including both task and dyad type as well as the interaction [*Log(BF_10_)* = 4.141]. A closer look at the analysis of effects across matched models revealed strong evidence in favour of the inclusion of task and moderate evidence in favour of the inclusion of the interaction as well as anecdotal evidence against the inclusion of dyad type as a predictor [task × dyad type: *Log(BF_incl_)* = 1.690; task: *Log(BF_incl_)* = 2.449; dyad type: *Log(BF_incl_)* = 0.049]. This seems to be driven by the increased difference between mixed and non-autistic dyads in the *meal planning* condition; although, in both conditions the silence-to-turn ratio was smaller in the case of non-autistic dyads.

##### 3.2.2.2. Turn-taking gap

Turn-taking gap was best explained by the model only including the predictor dyad type for which there was anecdotal evidence [*Log(BF_10_)* = 0.267]. Similarly, there was anecdotal evidence in favour of including dyad type as well as the interaction of dyad type and task but moderate evidence against including task [task × dyad type: *Log(BF_incl_)* = 0.877; task: *Log(BF_incl_)* = −1.369; dyad type: *Log(BF_incl_)* = 0.279]. Turn-taking gaps tended to be slightly longer in the mixed dyads, especially in the *meal planning* task.

##### 3.2.2.3. Pitch synchrony

Pitch synchrony, as calculated with WCLC, was best explained by the null model, suggesting that interactants of both dyad types adjusted their pitch to a similar extent to each other (see Supplementary material S5).

##### 3.2.2.4. Intensity synchrony

Nonetheless, non-autistic and mixed dyads differed in their WCLC synchrony of intensity with the best model predicting WLCL synchrony of intensity including both task and dyad type but not the interaction [*Log(BF_10_)* = 7.150]. Indeed, there is anecdotal evidence against the inclusion of the interaction, while there is decisive evidence for the inclusion of task and moderate evidence for the inclusion of dyad type [task × dyad type: *Log(BF_incl_)* = −0.813; task: *Log(BF_incl_)* = 5.567; dyad type: *Log(BF_incl_)* = 1.576]. Mixed dyads adjusted their intensity more strongly, with more synchrony in the *hobbies* condition in both dyad types.

##### 3.2.2.5. Comparison of dyads excluding male dyads

We repeated the analyses of silence-to-turn ratio, turn-taking gap, pitch synchrony and intensity synchrony in a limited sample excluding all male dyads to ensure that the found differences are not driven by differences in gender composition between mixed and non-autistic dyads. For all four parameters, the same model was supported by the evidence as the best model as for the full sample (see Supplementary material S6). Therefore, it is unlikely that the found differences were driven by gender composition.

## 4. Discussion

Differences in verbal communication are an important symptom of ASD (4, 7). We paired strangers and asked them to have two conversations, one about their hobbies and one where they collaboratively planned a meal with food and drinks that they both dislike. The dyads either consisted of two non-autistic adults or of one autistic and one non-autistic adult. This study aimed at answering the following research question: (i) what is the potential of speech and interactional features of communication for objective, reliable and scalable classification of ASD? Additionally, we used the extracted features to answer the following questions: (ii) how do autistic and non-autistic people differ with regards to their speech, and (iii) how do interactions between an autistic and a non-autistic person differ from interactions between two non-autistic people?

Regarding our main research question (i), we are able to present a multivariable prediction model that is able to distinguish between mixed and non-autistic dyads with above 75% of balanced accuracy. Automated extraction of speech and interactional features of verbal conversations offer an exciting new avenue for investigating symptoms as well as assisting the diagnosis of ASD. First, automated extraction increases objectivity and replicability while also providing a more detailed and fine-grained perspective on actual speech differences. This fine-grained perspective could in turn inform intervention by focusing on the specific aspects that differ between autistic and non-autistic conversation partners. Additionally, the current diagnostic procedures are time consuming, and recommendations include a combination of semi-structured interviews and neuropsychological assessments (64). This increases psychological stress for the affected person and their families (64, 65). Recent studies have shown that machine learning algorithms based on automatically extracted features could assist in this process (18, 27, 28, 53). Koehler et al. (28) automatically extracted movement parameters from the video recordings of the dyadic interactions analysed here, although in a slightly larger sample. A support vector machine based on the synchrony of facial expressions led to a balanced accuracy of almost 80% and a stacked model of different modalities achieved a balanced accuracy of 77.9%, both outperforming the here-proposed model. However, the extraction from speech and interactional features based on audio recordings offers an especially low-tech and user-friendly data collection procedure that is scalable and economic. As long as the environment is quiet and each of the interaction partners has their own microphone, the proposed preprocessing pipeline is easily applicable. Additionally, this study shows the feasibility of recording free conversations with predefined topics without the need of a semi-structured interview or of a trained conversation partner.

The here-presented and other studies (18, 27, 28, 53) show the potential of developing a multivariable prediction model to assist diagnostics of ASD. However, sample sizes in all of these studies are limited, and while they serve as a proof-of-concept, it is paramount to develop and validate such a multivariable prediction model with significantly larger sample sizes. Such a large-scale study could also compare different machine learning algorithms to ensure optimal performance. Automated extraction of speech and conversation features from audio recordings of people performing the *meal planning* task may be especially fruitful for collecting a large data set, especially if the here-presented effects persist in virtual conversations. Additionally, it is important to note that the autistic adults in our sample are not representative for many autistic adults, for example those with an intellectual disability or those who are non-verbal. This also limits the applicability of any developed prediction model based on speech and interactional features to a subsample of the autistic population. Furthermore, although the non-autistic sample did not differ from the autistic sample in age and gender distribution, a questionnaire measuring autism-like traits indicated that the non-autistic sample was positioned at one end and the autistic sample at the other end of this spectrum. Although this is representative of a non-clinical population, higher autism-like traits are also observed in other clinical populations (66). Future research should include a representative sample of other psychiatric diagnoses than ASD. This can only be achieved by evaluating the performance of the here-presented multivariable prediction model in a large-scale study to ensure its adequate translation to the clinical reality of the diagnostic process.

Concerning research question (ii), our results regarding the speech differences between autistic and non-autistic adults differ from a recent meta-analysis (5). While we found *increased* pitch and intensity variance as well as articulation rate in non-autistic compared to autistic adults, the authors of the meta-analysis report *decreased* pitch variability for non-autistic compared to autistic people as well as no significant differences regarding intensity variability and speech rate. However, the meta-analysis included a wider sample ranging from infants to adults and several modes of speech production including conversations, narration, semi-standardised tests and crying. Focusing on an adult sample and a conversation paradigm, Ochi et al. (18) found a decrease in the standard deviation of intensity in autistic compared to non-autistic adults but no difference in speech rate. Additionally, Kaland et al. (67) also found a decrease in pitch range in autistic compared to non-autistic adults. However, autistic adults seem to show a larger pitch range or variability compared to non-autistic adults in less naturalistic contexts including the narrative subtext of an assessment scale (68), answering questions about pictures (69) and when asked to produce a phrase conveying specific emotions (70). Interestingly, Hubbard et al. (70) used produced emotional phrases to assess whether the emotion is recognisable. They found that while phrases produced by autistic adults were matched with the intended emotion more often, they were also perceived as sounding less natural. Therefore, it is possible that autistic adults exaggerate in artificial contexts more strongly than non-autistic adults, leading to less natural and, most importantly, less representative speech. This would explain the differences in pitch variability between more and less interactive speech paradigms and highlights that speech in monologues and interactive dialogues needs to be distinguished in order to contextualise decreased or increased pitch variability in ASD.

Despite several interventions aiming at improving verbal communication skills and turn-taking (71–73), there is little research on differences in interactional features of conversations including autistic people. In this study, we investigated the ratio of silence to turns as well as the duration of the gaps between turns to investigate research question (iii). We found that mixed dyads had a credibly higher ratio of silence to turns, especially when collaboratively planning a meal. This indicates that the amount they were silent was higher, and they were speaking less. This is in line with the findings by Ochi et al. (18). However, we only found anecdotal evidence in favour of a difference in turn-taking gaps between mixed and non-autistic dyads, while Ochi and colleagues reported a clear effect of credibly longer turn-taking gaps when the ADOS was conducted with autistic adults compared to non-autistic adults. This elongation of turn-taking gaps has also been reported for children taking the ADOS by Bone et al. (74). Additionally, they found that both less speaking time and longer turn-taking gaps correlated with ADOS severity, and that there was a significant difference in the length of turn-taking gaps between children who were diagnosed with ASD and those who were diagnosed with other developmental disorders. This discrepancy could be due to the conversation topics. In our studies, the difference in turn-taking gaps was smaller in the *hobbies* task which could suggest that differences are reduced when autistic adults are talking about their special interests.

It is important to note that studies have shown that some differences in interactions can be reduced or even diminished when autistic individuals are interacting with other autistic people [75–80; for a possible theory explaining this phenomenon see Milton, (81)]. Since this study did not include dyads consisting of two autistic people, it is unclear if the found differences would extend to such a scenario. Future research examining interactional features in verbal communication should investigate possible differences not only between mixed and non-autistic dyads, but should also include comparisons with dyads consisting of two autistic interaction partners.

In addition to interactional features of verbal conversations, we also assessed synchrony and turn-based adaptation of speech features between two interaction partners in a dyad. We found no difference in turn-based adaptation between autistic and non-autistic adults, meaning that the extent to which they adapted their pitch, intensity and articulation rate to the previous turn was comparable in both groups. Similarly, we also did not find any differences between time-course synchrony of pitch between mixed and non-autistic dyads. However, we found that time-course synchrony of intensity was higher in mixed dyads than in non-autistic dyads. This is in contrast to Ochi et al. (18) who found increased correlation of the blockwise mean of intensity in conversations with non-autistic compared to autistic adults in the context of the ADOS. They also found a trend towards increased correlation of the blockwise mean of pitch in the conversations with non-autistic adults. Similarly, Lahiri et al. (27) found decreased dissimilarity of prosodic features which suggests increased synchrony in non-autistic children when analysing conversations from the ADOS. This is also more in line with previous research on other modalities which consistently shows reduced interpersonal synchrony in mixed dyads including an autistic person compared to dyads consisting of two non-autistic people (21). More research is needed to assess in which context interpersonal synchrony of speech features differs between autistic and non-autistic adults or mixed and non-autistic dyads.

Despite the insights this study offers, it is still unclear how context influences speech production with respect to ASD. We aimed for a naturalistic conversation setting with one common (*hobbies*) and one uncommon (*meal planning*) conversation topic. Other studies have opted to focus on a more controlled speech production by pairing participants with a trained diagnostician (18, 27), asking participants to retell a story (68) or even to produce a specific phrase with the aim of conveying a predefined emotion (70). Some of these contexts may have led to the differences in the reported results. The influence of context could be investigated by combining a naturalistic conversation task with a more controlled speech production task. The first would allow to assess speech features in an interactive settings similar to everyday conversations, while the latter could provide a baseline for each participant. Additionally, the influence of the interaction partners themselves has not been investigated yet. In our study, all interaction partners were strangers before the experiment, and in other studies, the interaction partners were often part of the research team (18).

In this study, we investigated the potential of speech and interactional features of verbal communication for digitally assisted diagnostics. We used automatic feature extraction on two naturalistic 10-minute conversations between either two non-autistic strangers (non-autistic dyad) or one autistic and one non-autistic stranger (mixed dyad). We were able to classify between individuals from a non-autistic vs. from a mixed dyad based on these features with high accuracy which offers a low-tech, economic and scalable option for diagnostic classification. Additionally, we have shown differences in pitch and intensity variation as well as articulation rate between autistic and non-autistic adults and differences in silence-to-turn ratio, turn-taking gaps and time-course synchrony of intensity between non-autistic and mixed dyads. This study shows the potential of verbal markers for diagnostic classification of ASD and suggests multiple relevant features showing differences between autistic and non-autistic adults.

## Data availability statement

The raw data supporting the conclusions of this article will be made available by the authors, without undue reservation.

## Ethics statement

The studies involving humans were approved by Ethikkommission der Medizinischen Fakultät der Ludwig-Maximilians-Universität München. The studies were conducted in accordance with the local legislation and institutional requirements. The participants provided their written informed consent to participate in this study.

## Author contributions

IP: Conceptualization, Formal analysis, Investigation, Methodology, Software, Supervision, Visualization, Writing – original draft, Writing – review & editing. JK: Conceptualization, Data curation, Funding acquisition, Investigation, Methodology, Project–administration, Writing – review & editing. AN: Conceptualization, Data curation, Investigation, Methodology, Project–administration, Writing – review & editing. NK: Conceptualization, Methodology, Resources, Supervision, Writing – review & editing. CF-W: Conceptualization, Funding acquisition, Investigation, Methodology, Resources, Supervision, Writing – review & editing.

## Footnotes

^1^ https://osf.io/jhetr

^2^ https://github.com/neurominer-git/NeuroMiner_1.1

^3^ https://github.com/IreneSophia/MLSPE
