# Supplementary material for: Automated extraction of speech and turn-taking parameters in autism allows for diagnostic classification using a multivariable prediction model
Source: Front Psychiatry. 2023 Nov 6;14:1257569. doi: 10.3389/fpsyt.2023.1257569 (PMC10658003; doi:10.3389/fpsyt.2023.1257569)
Supplement: Supplementary file 1 [file Data_Sheet_1.PDF]

Supplementary Material

1 Methods

1.1 Dyad comparison

1.1.1 Age: dyad mean and difference between interaction partners

Bayesian Mann-Whitney U Test

	$Log(BF_{10})$	W	Rhat
age_diff	-0.569	227.500	1.000
age_avg	-0.573	225.500	1.003

Note. Result based on data augmentation algorithm with 5 chains of 10000 iterations.

Descriptives

							95% Credible Interval	
	Group	N	Mean	SD	SE	Coefficient of variation	Lower	Upper
age_diff	mixed	26	12.692	9.177	1.800	0.723	8.986	16.399
	non-autistic	14	10.643	11.147	2.979	1.047	4.207	17.079
age_avg	mixed	26	33.154	7.720	1.514	0.233	30.036	36.272
	non-autistic	14	30.179	8.217	2.196	0.272	25.434	34.923

1.1.2 Gender composition

Contingency Tables

		gen_com			
dyadtype		female	male	mixed	Total
mixed	Count	4.000	9.000	13.000	26.000
	% within row	15.385 %	34.615 %	50.000 %	100.000 %
non-autistic	Count	7.000	0.000	7.000	14.000
	% within row	50.000 %	0.000 %	50.000 %	100.000 %
Total	Count	11.000	9.000	20.000	40.000
	% within row	27.500 %	22.500 %	50.000 %	100.000 %

Bayesian Contingency Tables Tests

	Value
Log (BF_{10}) Independent multinomial	2.692
N	40

Note. Proportion test restricted to 2 x 2 tables

1.2 Task setup

The interactions were recorded in two rooms for both of which the windows were completely covered, and the same stable lighting conditions were assured for each of the recordings. Interaction partners were seated approximately 190cm apart at a table. Three Logitech C922 cameras were connected to the same laptop (red in **Supplementary Figure** 1). Two of the cameras were mounted on small tripods and put on the table in front of the interaction partners so that each captured the facial expressions of one of the interaction partners. The third camera was installed on a larger tripod approximately 240cm from the middle of the table (A in **Supplementary Figure** 1). This camera was positioned to capture the full scene of the interaction, showing the interaction partners from the side. The recordings of all three cameras were automatically started with a PsychoPy script assuring time-locked video recordings. After the interaction partners were seated at the table, the experimenter attached the wristbands measuring heart rate and electrodermal activity (Empatica E4, yellow in **Supplementary Figure** 1) as well as the microphones (t.Bone earmic 500, blue in **Supplementary Figure** 1). Each interaction partner was fitted with their own microphone, recorded by the same recorder (ZoomH4n recorder, B in **Supplementary Figure** 1) as two separate channels in the same data file. The experimenter instructed the interaction partners to set a trigger on the wristbands. These triggers are accompanied by a blue light that was captured by the cameras allowing to time-lock the wristband data with the cameras. After the task was explained to the interaction partners, the experimenter used a movie clap board in front of the scene camera. The clap served as the time-stamp to time-lock the audio and video recordings.

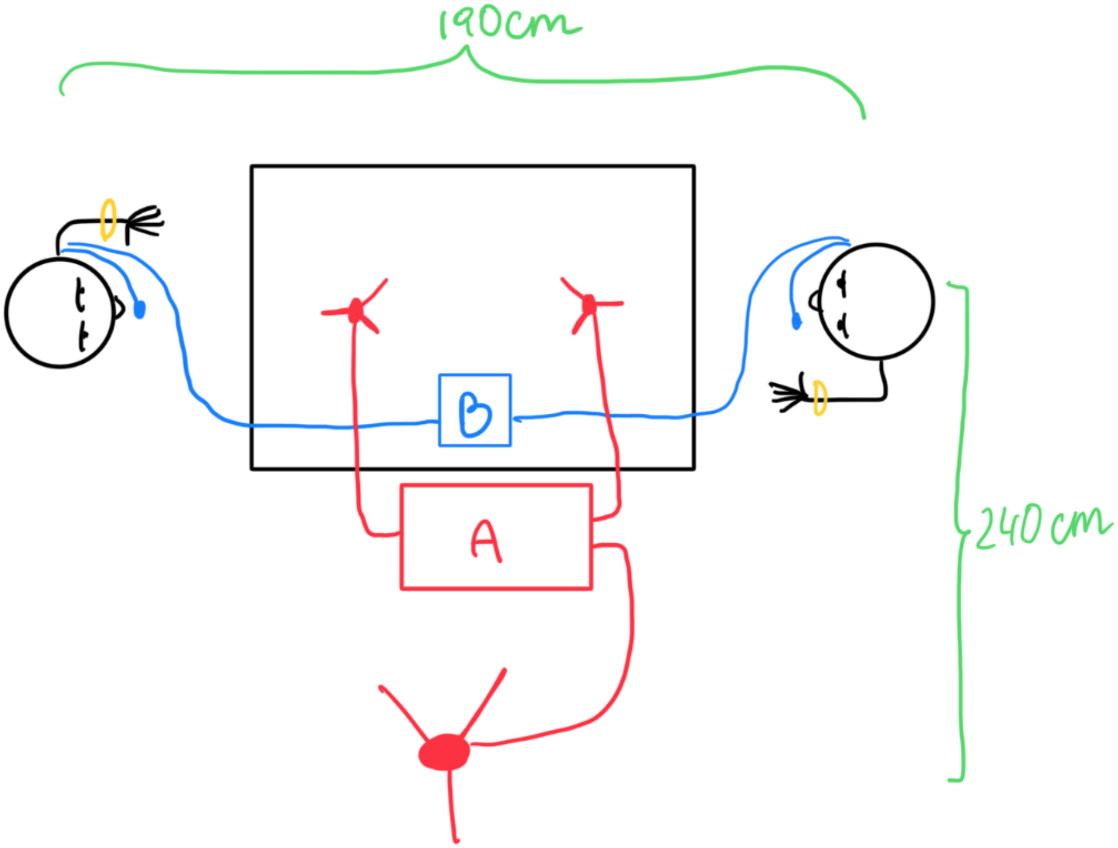

Supplementary Figure 1: Scheme of the setup of the conversation task.

1.3 Preprocessing pipeline

All scripts used in this preprocessing pipeline can be found on Github: https://github.com/IreneSophia/MLSPE. All audio data was aurally and visually inspected to ensure that the full ten minutes of each task have been recorded with sufficient quality in separate audio channels. All audio data of sufficient quality was further preprocessed in *praat* (Boersma and Weenink, 2022). We extracted minimum, maximum, mean and variance of pitch and intensity for each task and participant.

1.1.3 Pitch

We used *praat*'s autocorrelation method to extract pitch which has been shown to be reliable and accurate (Boersma and Weenink, 2022). The autocorrelation method has first been described by Boersma (1993), and requires the following parameters: frame duration or time step, pitch floor and ceiling determining the lower and upper bound of considered frequencies respectively, parameters determining silence and voicing thresholds and parameters determining favouring of high frequencies and disfavouring of pitch changes as well as transitions (octave cost, octave-jump cost and voiced/devoiced cost). We extracted pitch using the two-step method by Hirst (2011): first, we chose a low pitch floor of 50Hz and a high pitch ceiling of 700Hz in combination of a time step of 15ms to allow for a wide range of frequencies to be considered. For all other parameters, we used praat's reasonable default values. Second, we used the resulting pitch values to determine the first and third quartile of pitch values for each participant and task. Then, we computed individual pitch floors and ceilings with the following algorithm:

$$pitchfloor = min\left(0.75 \cdot Q1_{hobbies}, 0.75 \cdot Q1_{mealplanning}\right)$$

$$pitchceiling = max\left(2.5 \cdot Q3_{hobbies}, 2.5 \cdot Q3_{mealplanning}\right)$$

These individualised pitch floors (*range* = 46-168, *mean* = 107.5 ± 0.39 *SE*) and ceilings (*range* = 263-781, *mean* = 474.1 ± 1.85 *SE*) were then used to extract pitch information. To ensure that we considered the same number of frames per participant, we used the same time step for all participants. By default, *praat* determines the time step based on the following formula:

$$timestep = \frac{0.75}{pitchfloor}$$

Therefore, we determined the minimum of all individualised pitch floors to calculate the time step for all participants based on the same formula and set a time step of 0.016.

1.1.4 Intensity

We used *praat*'s algorithm to extract intensity (Boersma and Weenink, 2022) which convolves the squared sound with a Gaussian analysis window. It requires two parameters: minimum pitch referring to the minimum periodicity frequency of the signal and a time step. We chose the reasonable default value for pitch minimum, which is 100Hz, and a time step of 0.01s.

1.1.5 Continuous pitch and intensity

To estimate synchrony values, we determined pitch and intensity for each millisecond of recording resulting in continuous pitch and intensity time series. Since the analysis width depends on the pitch floor in the case of pitch, we did not use individual parameters but used the same parameters for all participants. In the case of pitch, we chose a wide range of considered frequencies with a pitch floor of 50Hz and a pitch ceiling of 700Hz due to the heterogeneity of our sample. In the case of intensity, we again used the default values provided by

praat. In the case of turn-based synchronisation, we correlated median pitch or intensity of each turn with the median pitch or intensity of the preceding turn.

1.1.6 Prosodic and turn-taking features

Next, we used the *uhm-o-meter* provided by De Jong and colleagues (2021; 2021) to differentiate between periods of speaking and silence as well as determine syllables and extract prosodic features (total number of syllables, total number of silent phases, duration of speaking as phonation time, speech rate as number of syllables per second, articulation rate as number of syllables per phonation time, average syllable duration and silence-to-turn ratio). The resulting speaking and silent instances were visually and aurally inspected for accuracy of categorisation. The labelling for eight tasks were judged as incorrect and excluded from all turn-based analysis. In the case of dyadic analyses, the respective interaction partners were excluded as well.

1.1.7 References

Boersma, P. (1993) 'Accurate short-term analysis of the fundamental frequency and the harmonics-to-noise ratio of a sampled sound', *Proceedings of the institute of phonetic sciences*, 17(1193), pp. 97–110.

Boersma, P. and Weenink, D. (2022) 'Praat: doing phonetics by computer'. Available at: http://www.praat.org/.

Hirst, D. (2011) 'The analysis by synthesis of speech melody: From data to models', *Journal of Speech Sciences*, 1(1), pp. 55–83. Available at: http://www.journalofspeechsciences.org/index.php/journalofspeechsciences/article/view/21.

De Jong, N. H., Pacilly, Jos and Heeren, W. (2021) 'PRAAT scripts to measure speed fluency and breakdown fluency in speech automatically', *Assessment in Education: Principles, Policy and Practice*. Routledge, 28(4), pp. 456–476. doi: 10.1080/0969594X.2021.1951162.

De Jong, N. H., Pacilly, J. and Heeren, W. (2021) 'uhm-o-meter [Computer software]'. Available at: https://sites.google.com/view/uhm-o-meter/home.

2 Details of the SVM classifier

Table S*1*

List of feature abbreviations

Individual	Abbreviation	Dyadic	Abbreviation
Articulation rate	art	Number of turns	dyad_no_turns
Number of pauses	npause	Silence-to-turn ratio	dyad_str
Number of syllables	nsyll	Speech rate	dyad_spr
Phonation time	pho	Synchrony of intensity	dyad_int_sync_ME A
Turn-based adaptation of art	art_sync	Synchrony of pitch	dyad_pit_sync_ME A
Turn-based adaptation of intensity	int_sync	Turn-taking gap	dyad_ttg
Turn-based adaptation of pitch	pit_sync		
Variance of intensity	int_var		
Variance of pitch	pit_var		

Note. All features were entered for the *meal planning* and the *hobbies* task separately, resulting in 30 features. The task is added to the abbreviation after an underscore. Articulation rate refers to the number of syllables per phonation time, while speech rate refers to the number of syllables per total time (phonation time and silence).

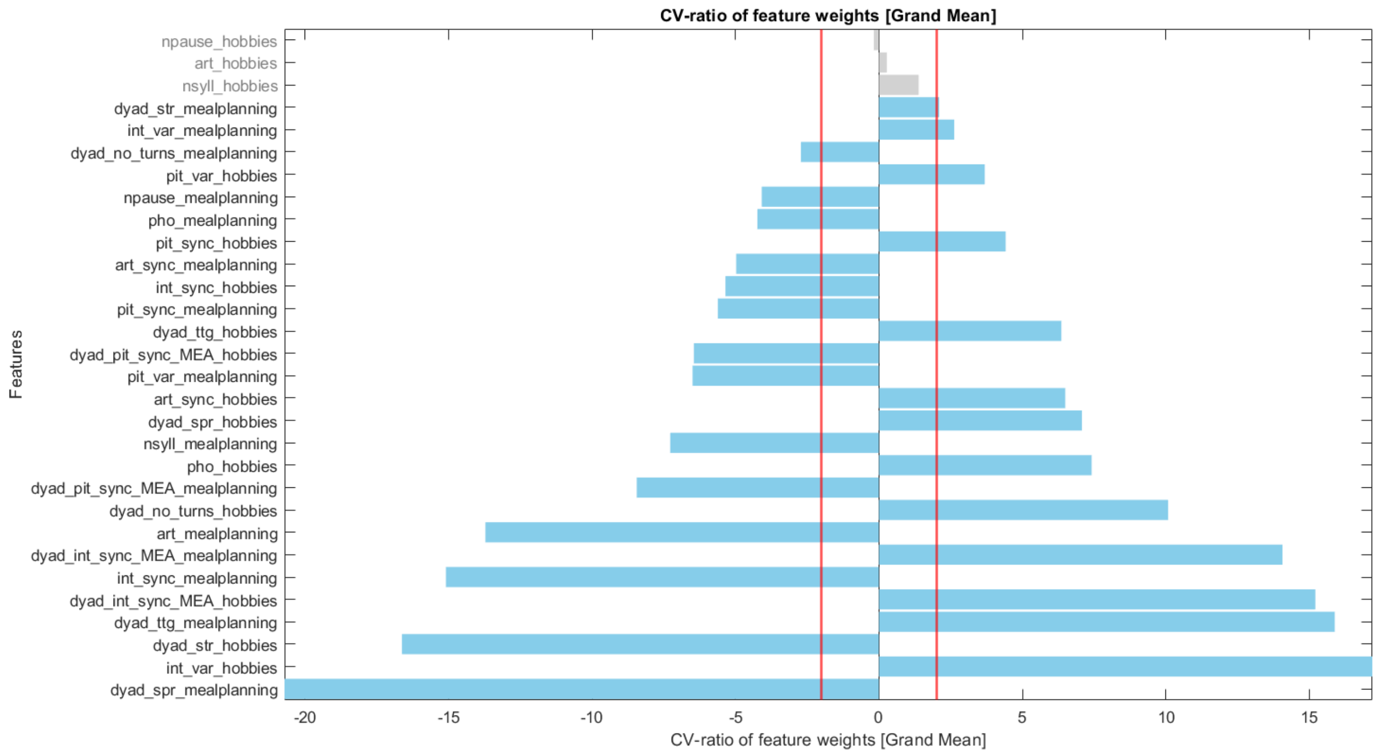

Supplementary Figure 2. Grand mean of the cross-validation ratio for each of the feature weights.

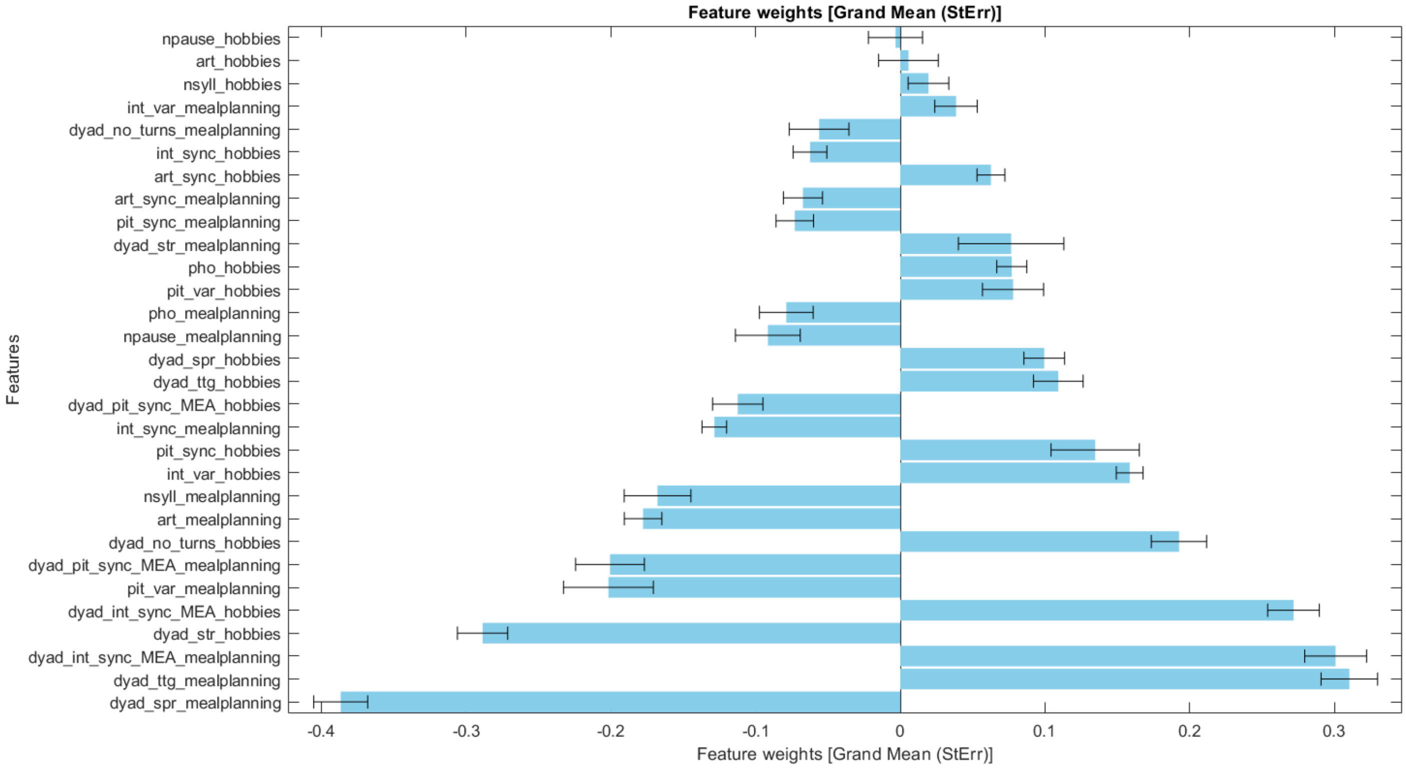

Supplementary Figure 3. Grand mean and standard error of the feature weights.

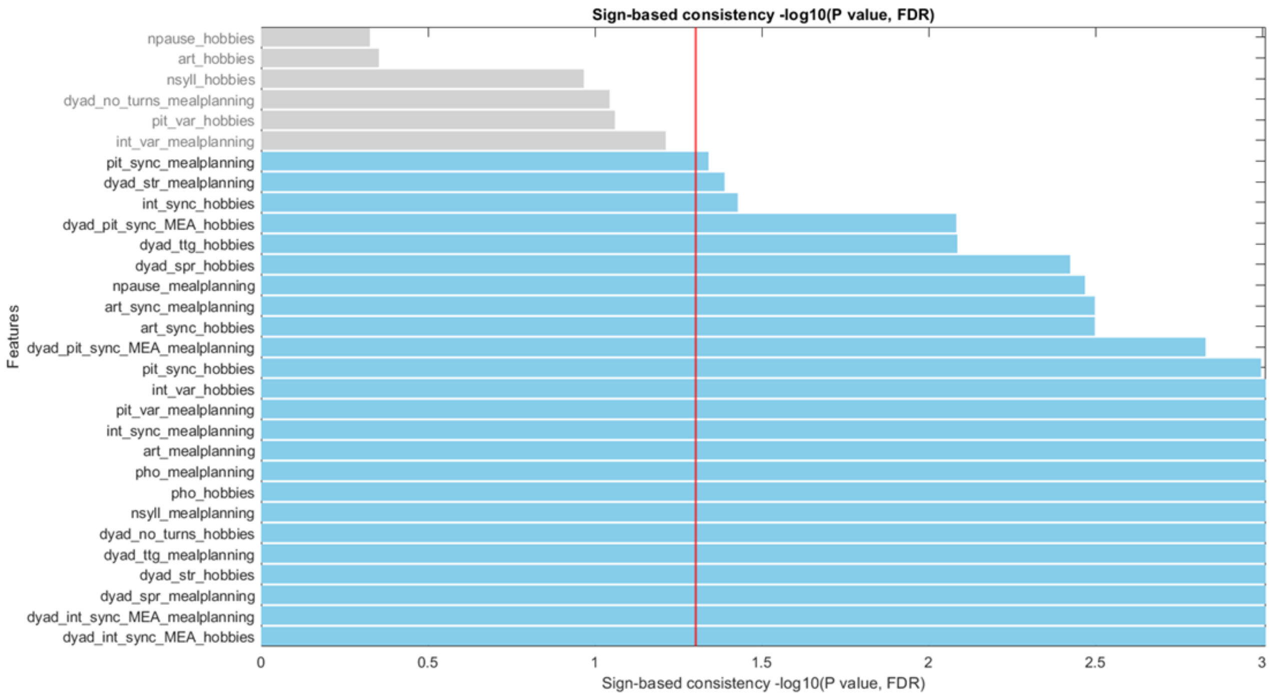

Supplementary Figure 4. Negative log *p*-value (corrected with false discovery rate) to show sign-based consistency.

S3: Pseudosynchrony and pseudoadaptation for ML speech

I. S. Plank

03/01/2023

General information

This R Markdown document analyses pseudosynchronisation in the speech data of the ML study. To run this script, one needs the following file:

- “ML_turns.csv” which contains all turn information needed for section sliding
- “ML_indi.csv” which contains the actual synchronisation values for comparison
- “ML_dyad.csv” which contains the actual synchrony values for comparison

Software versions

```
R.Version()$version.string
```

```
## [1] "R version 4.3.0 (2023-04-21)"
```

```
packageVersion('ggpubr')
```

```
## [1] '0.6.0'
```

```
packageVersion('ggsignif')
```

```
## [1] '0.6.4'
```

```
packageVersion('tidyverse')
```

```
## [1] '2.0.0'
```

```
packageVersion('knitr')
```

```
## [1] '1.43'
```

```
packageVersion('BayesFactor')
```

```
## [1] '0.9.12.4.4'
```

```
packageVersion('rMEA')
```

```
## [1] '1.2.2'
```

```
packageVersion('pastecs')
```

```
## [1] '1.3.21'
```

Individual level: turn-based pseudosynchronisation

For turn-based pseudosynchronisation, this script uses an approach described in Moulder et al. (2018, *Psychol Methods*) to test the following hypothesis: “Synchrony does not exist between sections of size *m* in these two time series” (section sliding). This segment shuffling, from Moulder et al., “requires researchers to cut a time series *X* into shorter sections of size *m* which are randomly appended to one another to create a new time series *Xs* until no section is in its original position”. This new time series is then paired with an unchanged time series *Y*. Since the synchronisation is already based on sections or speech turns in the conversation, no data shuffling will be performed. Since each dyad can have a unique amount of turns, no dyad shuffling will be performed.

For each speaker in each task, the order of the previous turns are shuffled 100 times while the order of the current turns remain unchanged. Note that no check for uniqueness is performed. In the end, an average for this specific speaker and task is calculated from the 100 pseudosynchrony values.

Segment shuffling: is there synchrony between turns

in these two time series?

```
filename = "ML_indi_st-shuffle.RData"

if (!file.exists(filename)) {

  speaker  = c()
  psyncint = c()
  psyncpit = c()
  psyncart = c()

  df = read_csv("ML_turns.csv", show_col_types = F) %>%
    mutate(speaker = paste(name, speaker, sep = "_")) %>%
    select(c(speaker, pit_turn, pit_prev_turn,
             int_turn, int_prev_turn,
             art_prev_turn, art_turn))

  for (s in unique(df$speaker)) {
    speaker = c(speaker, rep(s, times = n))
    df.sel = df %>% filter(speaker == s)
    for (i in 1:n) {
      # shuffling
      df.sel$pit_prev  = sample(df.sel$pit_prev_turn)
      df.sel$int_prev  = sample(df.sel$int_prev_turn)
      df.sel$art_prev = sample(df.sel$art_prev_turn)
      psyncint = c(psyncint, abs(cor(df.sel$int_turn,df.sel$int_prev, use = "na.or.complete")))
      psyncpit = c(psyncpit, abs(cor(df.sel$pit_turn,df.sel$pit_prev, use = "na.or.complete")))
      psyncart = c(psyncart, abs(cor(df.sel$art_turn,df.sel$art_prev, use = "na.or.complete")))
    }
  }
  df.s_shuffle = data.frame(speaker,psyncint, psyncpit, psyncart)
  save(df.s_shuffle,file=filename)
} else {
  load(filename)
}
```

```
df.sync = read_csv("ML_indi.csv", show_col_types = F) %>%
  separate(
    subject, c("ML", "dyad", "speaker")
  ) %>%
  mutate(
    speaker = paste(ML, dyad, task, speaker, sep = "_")
  ) %>%
  select(c(speaker, pit_sync, int_sync, art_sync))

df.agg  = df.s_shuffle %>% group_by(speaker) %>%
  summarise(
    pit_psync  = mean(psyncpit),
    int_psync  = mean(psyncint),
    art_psync  = mean(psyncart)
  )

df.agg = merge(df.sync,df.agg)

kable(stat.desc(df.agg %>% select(where(is.numeric)) %>% na.omit()))
```

	pit_sync	int_sync	art_sync	pit_psync	int_psync	art_psync
nbr.val	146.0000000	146.0000000	146.0000000	146.0000000	146.0000000	146.0000000
nbr.null	0.0000000	0.0000000	0.0000000	0.0000000	0.0000000	0.0000000
nbr.na	0.0000000	0.0000000	0.0000000	0.0000000	0.0000000	0.0000000
min	0.0000473	0.0010045	0.0001826	0.0354163	0.0528357	0.0640312
max	0.6216985	0.3861174	0.4167157	0.1523578	0.1394368	0.1639475
range	0.6216512	0.3851130	0.4165330	0.1169415	0.0866011	0.0999163
sum	17.5995630	21.2777387	20.0971049	12.7742778	13.2213065	14.4787928
median	0.0954132	0.1392675	0.1250022	0.0855785	0.0881951	0.0959025
mean	0.1205450	0.1457379	0.1376514	0.0874951	0.0905569	0.0991698
SE.mean	0.0088649	0.0070925	0.0079585	0.0016388	0.0014305	0.0016067
CI.mean.0.95	0.0175211	0.0140180	0.0157296	0.0032390	0.0028274	0.0031756
var	0.0114736	0.0073443	0.0092473	0.0003921	0.0002988	0.0003769
std.dev	0.1071150	0.0856989	0.0961627	0.0198018	0.0172852	0.0194138
coef.var	0.8885899	0.5880343	0.6985959	0.2263191	0.1908770	0.1957636

```
res = ttestBF(x = df.agg[!is.na(df.agg$pit_sync),]$pit_sync,
        y = df.agg[!is.na(df.agg$pit_sync),]$pit_psync,
        paired = T)
res
```

```
## Bayes factor analysis
## --------------
## [1] Alt., r=0.707 : 95.68174 ±0%
##
## Against denominator:
##   Null, mu = 0
## ---
## Bayes factor type: BFoneSample, JZS
```

```
res@bayesFactor[["bf"]]
```

```
## [1] 4.561027
```

```
res = ttestBF(x = df.agg[!is.na(df.agg$int_sync),]$int_sync,
        y = df.agg[!is.na(df.agg$int_sync),]$int_psync,
        paired = T)
res
```

```
## Bayes factor analysis
## --------------
## [1] Alt., r=0.707 : 1289385219 ±0%
##
## Against denominator:
##   Null, mu = 0
## ---
## Bayes factor type: BFoneSample, JZS
```

```
res@bayesFactor[["bf"]]
```

```
## [1] 20.97743
```

```
res = ttestBF(x = df.agg[!is.na(df.agg$art_sync),]$art_sync,
        y = df.agg[!is.na(df.agg$art_sync),]$art_psync,
        paired = T)
res
```

```
## Bayes factor analysis
## --------------
## [1] Alt., r=0.707 : 1114.939 ±0%
##
## Against denominator:
##   Null, mu = 0
## ---
## Bayes factor type: BFoneSample, JZS
```

```
res@bayesFactor[["bf"]]
```

```
## [1] 7.016555
```

The t-tests show that there is a significant difference between the pseudosynchronisation values obtained through segment shuffling and the real synchronisation values in the case of turn-based synchronisation of intensity, pitch and articulation rate, with the real synchronisation values being higher.

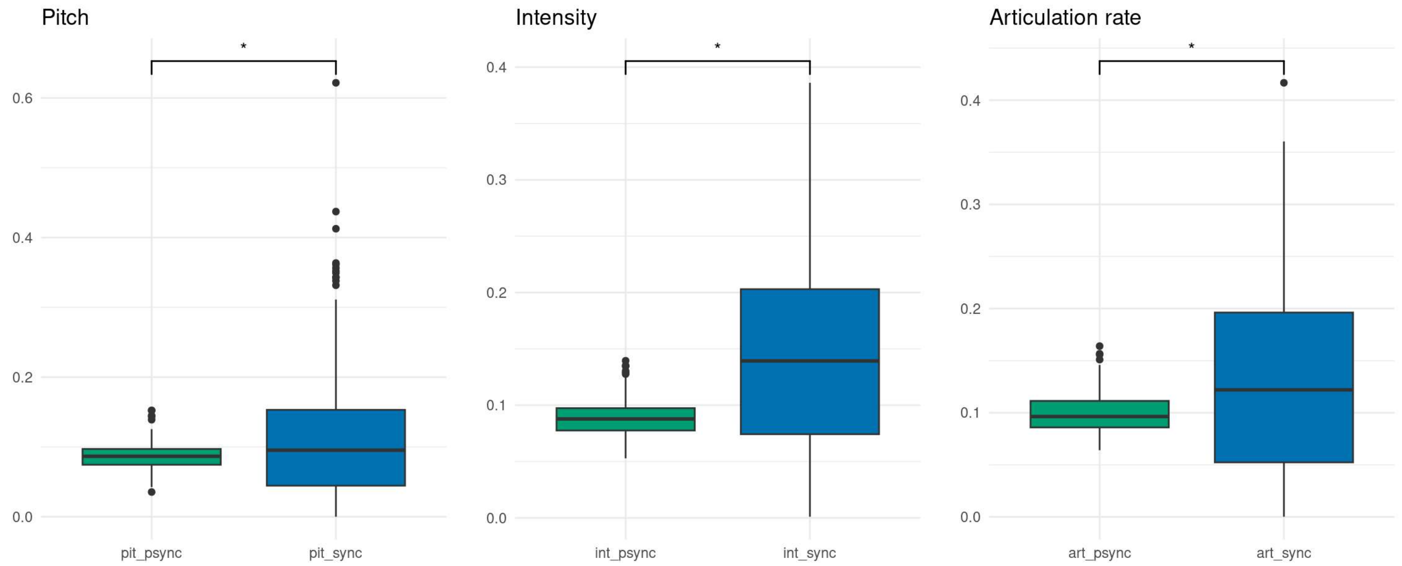

Dyad level: timecourse pseudosynchrony based on CCF

We again use segment shuffling to compare pseudosynchrony and synchrony values on the dyad level. Pitch and intensity synchrony was calculated using the CCF function from rMEA. Therefore, we use the same function with the same window size but with shuffled windows for calculating pseudosynchrony.

For each speaker in each task, their time series is shuffled 50 times and then paired with the unchanged time series of their partner in the dyad. Note that no check for uniqueness is performed. In the end, an average for this specific dyad and task is calculated from the 100 pseudosynchrony values.

Segment shuffling: is there synchrony between sections of 16 seconds (pitch, intensity) in these two

time series?

```
filename = "ML_dyad_s-shuffle.RData"

if (!file.exists(filename)) {

  # create new data frame for pseudosync values
  cols = c("dyad", "dyad type", "task", "pit_psync_MEA", "int_psync_MEA")
  df.psync = data.frame(matrix(nrow = 0, ncol = length(cols)))
  colnames(df.psync) = cols

  # load actual sync values
  df.dyad = read_csv("ML_dyad.csv", show_col_types = F)

  sr = 100

  # go through all dyads
  for (i in 1:nrow(df.dyad)) {
    # load the respective data of continuous pitch and intensity
    ptn = paste("^ch_.*", df.dyad$dyad[i], ".*", df.dyad$task[i], ".*_cont.csv", sep = "")
    fls = list.files(pattern = ptn)
    df_L = read_delim(fls[1], delim = ";", show_col_types = F, col_types = cols(.default = "d"))
    df_R = read_delim(fls[2], delim = ";", show_col_types = F, col_types = cols(.default = "d"))
    # resample to 100Hz
    L_pit = df_L$pitch
    L_pit[is.na(L_pit)] = 0
    L_pit = signal::resample(L_pit,1,1000/sr)
    L_int = df_L$int
    L_int[is.na(L_int)] = 0
    L_int = signal::resample(L_int,1,1000/sr)
    R_pit = df_R$pitch
    R_pit[is.na(R_pit)] = 0
    R_pit = signal::resample(R_pit,1,1000/sr)
    R_int = df_R$int
    R_int[is.na(R_int)] = 0
    R_int = signal::resample(R_int,1,1000/sr)
    # create fakeMEA and divide into segments
    mea_int  = fakeMEA(L_int,R_int,sr,"int_L","int_R")
    mea_pit  = fakeMEA(L_pit,R_pit,sr,"pit_L","pit_R")
    mea_int_L = split(mea_int[["all_01_01"]][["MEA"]][["s1Name"]], floor(seq_along(mea_int[["all
_01_01"]][["MEA"]][["s1Name"]])/16))
    mea_int_R = split(mea_int[["all_01_01"]][["MEA"]][["s2Name"]], floor(seq_along(mea_int[["all
_01_01"]][["MEA"]][["s2Name"]])/16))
    mea_pit_L = split(mea_pit[["all_01_01"]][["MEA"]][["s2Name"]], floor(seq_along(mea_pit[["all
_01_01"]][["MEA"]][["s1Name"]])/16))
    mea_pit_R = split(mea_pit[["all_01_01"]][["MEA"]][["s2Name"]], floor(seq_along(mea_pit[["all
_01_01"]][["MEA"]][["s2Name"]])/16))
    # shuffle each side n/2 times
    for (j in 1:(n/2)) {

      # shuffling left
```

```
      mea_int[["all_01_01"]][["MEA"]][,2] = unlist(mea_int_R)
      mea_int[["all_01_01"]][["MEA"]][,1] = unlist(sample(mea_int_L))
      mea_int = MEAccf(mea_int,2,16,8)
      mea_pit[["all_01_01"]][["MEA"]][,2] = unlist(mea_pit_R)
      mea_pit[["all_01_01"]][["MEA"]][,1] = unlist(sample(mea_pit_L))
      mea_pit = MEAccf(mea_pit,2,16,8)
      # create new row with mean of peaks of windows
      new = c(df.dyad$dyad[i],
              df.dyad$`dyad type`[i],
              df.dyad$task[i],
              mean(apply(mea_int$all_01_01$ccf,1,max)),
              mean(apply(mea_pit$all_01_01$ccf,1,max)))
      df.psync[nrow(df.psync) + 1,] = new

      # shuffling right
      mea_int[["all_01_01"]][["MEA"]][,2] = unlist(sample(mea_int_R))
      mea_int[["all_01_01"]][["MEA"]][,1] = unlist(mea_int_L)
      mea_int = MEAccf(mea_int,2,16,8)
      mea_pit[["all_01_01"]][["MEA"]][,2] = unlist(sample(mea_pit_R))
      mea_pit[["all_01_01"]][["MEA"]][,1] = unlist(mea_pit_L)
      mea_pit = MEAccf(mea_pit,2,16,8)
      # create new row with mean of peaks of windows
      new = c(df.dyad$dyad[i],
              df.dyad$`dyad type`[i],
              df.dyad$task[i],
              mean(apply(mea_int$all_01_01$ccf,1,max)),
              mean(apply(mea_pit$all_01_01$ccf,1,max)))
      df.psync[nrow(df.psync) + 1,] = new
    }
  }
  # save everything
  save(df.psync,file=filename)
} else {
  load(filename)
}
```

```
df.sync = read_csv("ML_dyad.csv", show_col_types = F) %>%
  select(c(dyad, `dyad type`, task, pit_sync_MEA, int_sync_MEA))

df.agg  = df.psync %>%
  mutate(
    pit_psync_MEA = as.numeric(pit_psync_MEA),
    int_psync_MEA = as.numeric(int_psync_MEA)
  ) %>%
  group_by(dyad, task) %>%
  summarise(
    pit_psync_MEA  = mean(pit_psync_MEA),
    int_psync_MEA  = mean(int_psync_MEA)
  )

df.agg = merge(df.sync,df.agg)

kable(stat.desc(df.agg %>% select(where(is.numeric)) %>% na.omit()))
```

	pit_sync_MEA	int_sync_MEA	pit_psync_MEA	int_psync_MEA
nbr.val	73.0000000	73.0000000	73.0000000	73.0000000
nbr.null	0.0000000	0.0000000	0.0000000	0.0000000
nbr.na	0.0000000	0.0000000	0.0000000	0.0000000
min	0.1508441	0.2854091	0.1822206	0.1467557
max	0.2600965	0.4852903	0.1968212	0.1955330
range	0.1092524	0.1998812	0.0146006	0.0487773
sum	14.3970550	26.8665634	13.8869142	11.9498496
median	0.1951318	0.3665821	0.1900176	0.1630618
mean	0.1972199	0.3680351	0.1902317	0.1636966
SE.mean	0.0025798	0.0055779	0.0003829	0.0011814
CI.mean.0.95	0.0051427	0.0111194	0.0007634	0.0023551
var	0.0004858	0.0022713	0.0000107	0.0001019
std.dev	0.0220416	0.0476577	0.0032717	0.0100940
coef.var	0.1117613	0.1294923	0.0171987	0.0616631

```
res = ttestBF(x = df.agg[!is.na(df.agg$pit_sync_MEA),]$pit_sync_MEA,
        y = df.agg[!is.na(df.agg$pit_sync_MEA),]$pit_psync_MEA,
        paired = T)
res
```

```
## Bayes factor analysis
## --------------
## [1] Alt., r=0.707 : 4.149943 ±0.01%
##
## Against denominator:
##   Null, mu = 0
## ---
## Bayes factor type: BFoneSample, JZS
```

```
res@bayesFactor[["bf"]]
```

```
## [1] 1.423095
```

```
res = ttestBF(x = df.agg[!is.na(df.agg$int_sync_MEA),]$int_sync_MEA,
        y = df.agg[!is.na(df.agg$int_sync_MEA),]$int_psync_MEA,
        paired = T)
res
```

```
## Bayes factor analysis
## --------------
## [1] Alt., r=0.707 : 2.397301e+45 ±0%
##
## Against denominator:
##   Null, mu = 0
## ---
## Bayes factor type: BFoneSample, JZS
```

```
res@bayesFactor[["bf"]]
```

```
## [1] 104.4907
```

The t-tests show that there is a significant difference between the pseudosynchrony values obtained through segment shuffling and the real synchrony values in the case of MEA-based CCF synchrony of intensity and pitch, with the real synchrony values being higher.

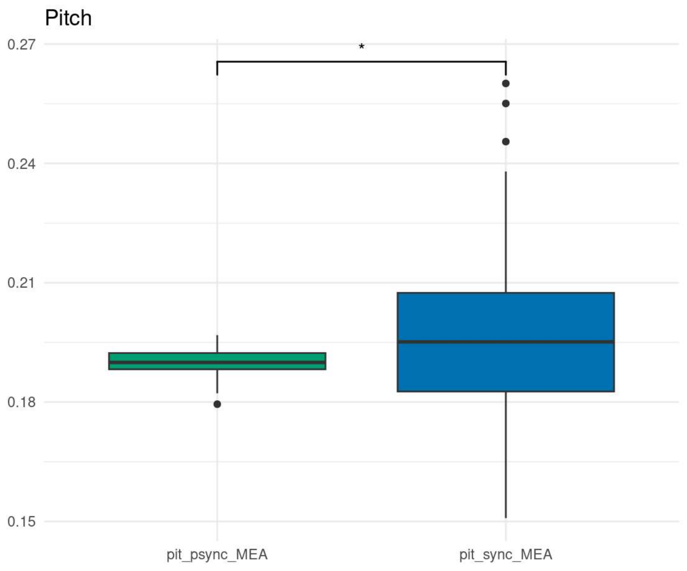
Pitch
*
0.27
0.24
0.21
0.18
0.15
pit_psync_MEA
pit_sync_MEA

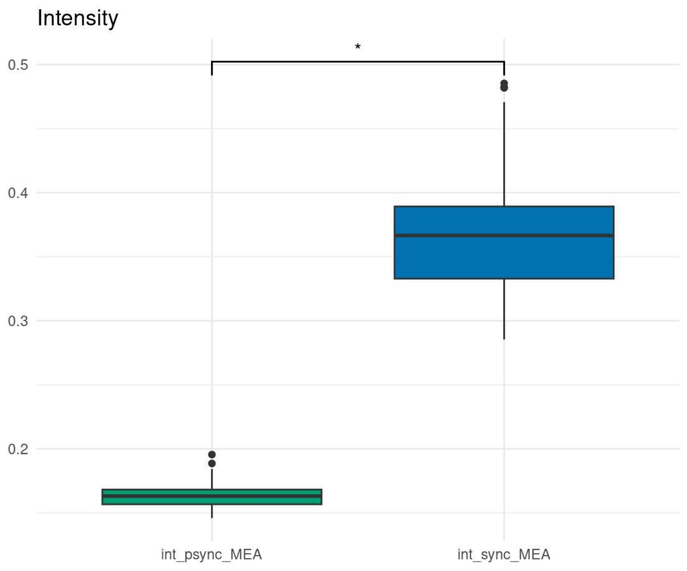
Intensity
*
0.5
0.4
0.3
0.2
int_psync_MEA
int_sync_MEA

S4: Results comparing individual participants

This JASP file contains all assumption checks and analyses to test the following hypotheses:

1. We hypothesise that there will be differences in pitch between ASD and TD participants, such that ASD participants will show less variance in pitch than TD participants.
2. We hypothesise that there will be differences in intensity between ASD and TD participants, such that ASD participants will show less variance in intensity than TD participants.
3. We hypothesise that there will be differences in articulation rate between ASD and TD participants, such that ASD participants will show a decreased articulation rate compared to TD participants.
4. We hypothesise that there will be differences in synchronisation of pitch between ASD and TD participants, such that ASD participants will show less synchronisation of pitch than TD participants.
5. We hypothesise that there will be differences in synchronisation of intensity between ASD and TD participants, such that ASD participants will show less synchronisation of intensity than TD participants.

We also hypothesised differences in synchronisation of articulation rate, however, it did not exceed the levels of pseudosynchrony.

pit_var: Bayesian Repeated Measures ANOVA

Model Comparison

Models	P(M)	P(M|data)	$Log(BF_M)$	$Log(BF_{10})$	error %
Null model (incl. subject and random slopes)	0.200	0.002	−4.904	0.000	
task + diagnostic status	0.200	0.507	1.413	5.612	2.179
task + diagnostic status + task ✻ diagnostic status	0.200	0.304	0.558	5.102	3.859
task	0.200	0.181	−0.121	4.585	1.864
diagnostic status	0.200	0.006	−3.699	1.201	5.605

Note. All models include subject, and random slopes for all repeated measures factors.

Analysis of Effects

Effects	P(incl)	P(excl)	P(incl|data)	P(excl|data)	$Log(BF_{incl})$
task	0.400	0.400	0.688	0.008	4.455
diagnostic status	0.400	0.400	0.513	0.183	1.030
task ✻ diagnostic status	0.200	0.200	0.304	0.507	−0.510

Note. Compares models that contain the effect to equivalent models stripped of the effect. Higher-order interactions are excluded. Analysis suggested by Sebastiaan Mathôt.

Model Averaged Q-Q Plot

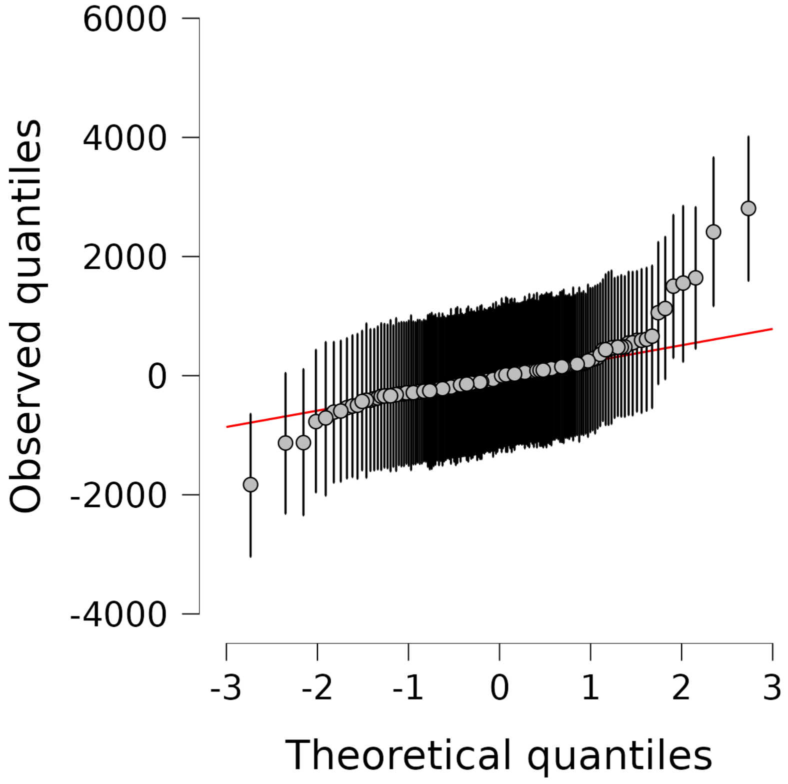

Descriptives

Descriptives

task	diagnostic status	N	Mean	SD	SE	Coefficient of variation
hobbies	ASD	26	930.856	973.995	191.016	1.046
	TD	54	1720.993	1492.267	203.072	0.867
mealplanning	ASD	26	1140.318	1420.396	278.563	1.246
	TD	54	2244.362	2202.542	299.728	0.981

int_var: Bayesian Repeated Measures ANOVA

Model Comparison

Models	P(M)	P(M|data)	$Log(BF_M)$	$Log(BF_{10})$	error %
Null model (incl. subject and random slopes)	0.200	0.038	−1.858	0.000	
task + diagnostic status + task ✻ diagnostic status	0.200	0.925	3.903	3.205	4.926
diagnostic status	0.200	0.024	−2.318	−0.446	3.641
task	0.200	0.008	−3.455	−1.566	2.067
task + diagnostic status	0.200	0.005	−3.849	−1.958	4.433

Note. All models include subject, and random slopes for all repeated measures factors.

Analysis of Effects

Effects	P(incl)	P(excl)	P(incl|data)	P(excl|data)	$Log(BF_{incl})$
task	0.400	0.400	0.013	0.062	−1.544
diagnostic status	0.400	0.400	0.029	0.045	−0.436
task ✻ diagnostic status	0.200	0.200	0.925	0.005	5.163

Note. Compares models that contain the effect to equivalent models stripped of the effect. Higher-order interactions are excluded. Analysis suggested by Sebastiaan Mathôt.

Model Averaged Q-Q Plot

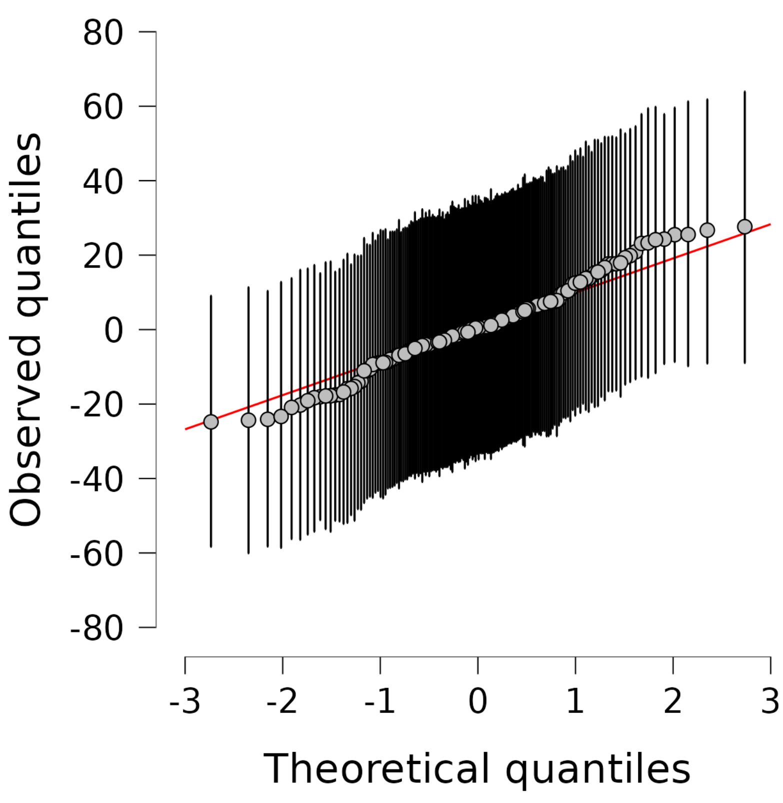

Descriptives

Descriptives

task	diagnostic status	N	Mean	SD	SE	Coefficient of variation
hobbies	ASD	26	170.943	75.906	14.886	0.444
	TD	54	169.608	66.304	9.023	0.391
mealplanning	ASD	26	158.373	75.819	14.869	0.479
	TD	54	178.378	65.195	8.872	0.365

Model Comparison

Models	P(M)	P(M|data)	$\text{Log}(BF_M)$	$\text{Log}(BF_{10})$	error %
Null model (incl. subject and random slopes)	0.200	0.001	−5.440	0.000	
task + diagnostic status + task ✻ diagnostic status	0.200	0.905	3.636	6.727	8.519
task + diagnostic status	0.200	0.073	−1.155	4.210	2.560
task	0.200	0.016	−2.728	2.696	0.903
diagnostic status	0.200	0.005	−3.873	1.563	1.995

Note. All models include subject, and random slopes for all repeated measures factors.

Analysis of Effects

Effects	P(incl)	P(excl)	P(incl|data)	P(excl|data)	$\text{Log}(BF_{incl})$
task	0.400	0.400	0.089	0.006	2.656
diagnostic status	0.400	0.400	0.078	0.017	1.517
task ✻ diagnostic status	0.200	0.200	0.905	0.073	2.517

Note. Compares models that contain the effect to equivalent models stripped of the effect. Higher-order interactions are excluded. Analysis suggested by Sebastiaan Mathôt.

Model Averaged Q-Q Plot

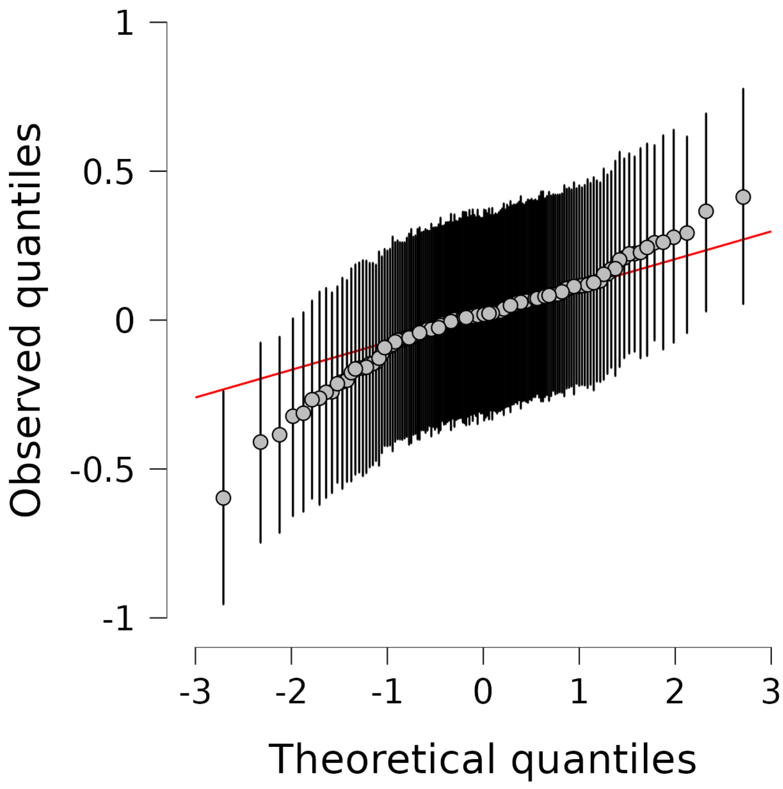

Descriptives

Descriptives

task	diagnostic status	N	Mean	SD	SE	Coefficient of variation
hobbies	ASD	24	3.963	0.395	0.081	0.100
	TD	50	4.155	0.473	0.067	0.114
mealplanning	ASD	24	3.715	0.514	0.105	0.138
	TD	50	4.116	0.465	0.066	0.113

pit_sync: Bayesian Repeated Measures ANOVA

Model Comparison

Models	P(M)	P(M|data)	$\log(BF_M)$	$\log(BF_{10})$	error %
Null model (incl. subject and random slopes)	0.200	0.613	1.847	0.000	
diagnostic status	0.200	0.153	−0.328	−1.391	0.815
task	0.200	0.142	−0.411	−1.462	1.407
task + diagnostic status + task ✻ diagnostic status	0.200	0.056	−1.441	−2.396	16.710
task + diagnostic status	0.200	0.036	−1.899	−2.833	1.610

Note. All models include subject, and random slopes for all repeated measures factors.

Analysis of Effects

Effects	P(incl)	P(excl)	P(incl|data)	P(excl|data)	$\log(BF_{incl})$
task	0.400	0.400	0.178	0.766	−1.458
diagnostic status	0.400	0.400	0.189	0.755	−1.387
task ✻ diagnostic status	0.200	0.200	0.056	0.036	0.437

Note. Compares models that contain the effect to equivalent models stripped of the effect. Higher-order interactions are excluded. Analysis suggested by Sebastiaan Mathôt.

Model Averaged Q-Q Plot

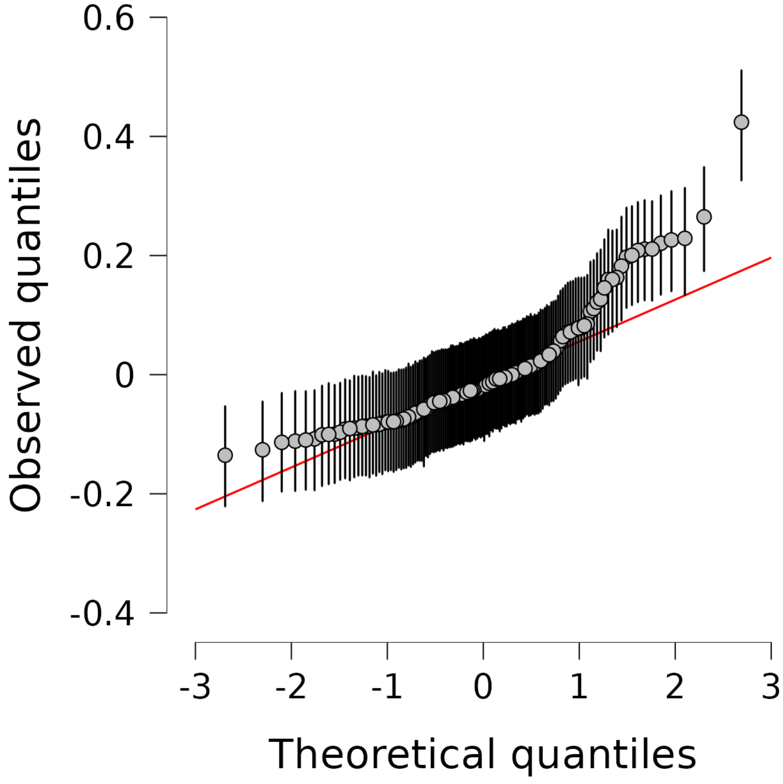

Descriptives

Descriptives

task	diagnostic status	N	Mean	SD	SE	Coefficient of variation
hobbies	ASD	21	0.163	0.108	0.024	0.662
	TD	49	0.115	0.124	0.018	1.077
mealplanning	ASD	21	0.099	0.071	0.016	0.720
	TD	49	0.125	0.102	0.015	0.815

int_sync: Bayesian Repeated Measures ANOVA

Model Comparison

Models	P(M)	P(M|data)	$\text{Log}(BF_M)$	$\text{Log}(BF_{10})$	error %
Null model (incl. subject and random slopes)	0.200	0.677	2.127	0.000	
diagnostic status	0.200	0.153	−0.326	−1.488	1.633
task	0.200	0.118	−0.623	−1.745	0.795
task + diagnostic status	0.200	0.027	−2.197	−3.221	1.793
task + diagnostic status + task ✻ diagnostic status	0.200	0.025	−2.285	−3.307	1.784

Note. All models include subject, and random slopes for all repeated measures factors.

Analysis of Effects

Effects	P(incl)	P(excl)	P(incl|data)	P(excl|data)	$\text{Log}(BF_{incl})$
task	0.400	0.400	0.145	0.830	−1.743
diagnostic status	0.400	0.400	0.180	0.795	−1.486
task ✻ diagnostic status	0.200	0.200	0.025	0.027	−0.086

Note. Compares models that contain the effect to equivalent models stripped of the effect. Higher-order interactions are excluded. Analysis suggested by Sebastiaan Mathôt.

Model Averaged Q-Q Plot

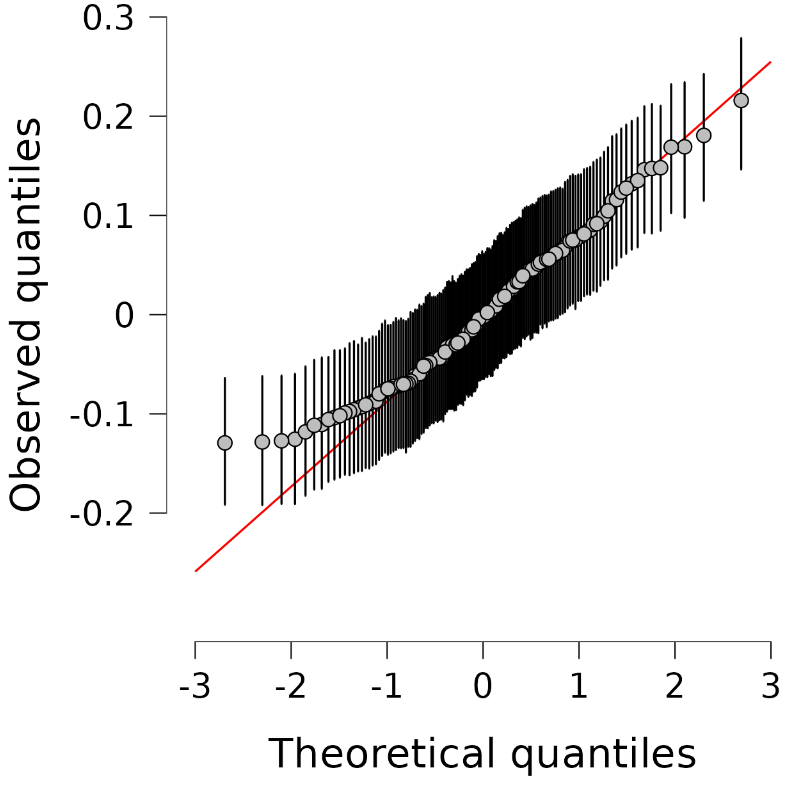

Descriptives

Descriptives

task	diagnostic status	N	Mean	SD	SE	Coefficient of variation
hobbies	ASD	21	0.164	0.093	0.020	0.565
	TD	49	0.137	0.085	0.012	0.617
mealplanning	ASD	21	0.129	0.093	0.020	0.717
	TD	49	0.154	0.082	0.012	0.530

art_sync: Bayesian Repeated Measures ANOVA

Model Comparison

Models	P(M)	P(M|data)	Log(BF_M)	Log(BF_{10})	error %
Null model (incl. subject and random slopes)	0.200	0.201	0.004	0.000	
task	0.200	0.586	1.734	1.072	5.255
task + diagnostic status	0.200	0.129	−0.522	−0.441	3.761
diagnostic status	0.200	0.043	−1.724	−1.547	1.074
task + diagnostic status + task ✻ diagnostic status	0.200	0.042	−1.753	−1.576	3.276

Note. All models include subject, and random slopes for all repeated measures factors.

Analysis of Effects

Effects	P(incl)	P(excl)	P(incl|data)	P(excl|data)	Log(BF_{incl})
task	0.400	0.400	0.715	0.243	1.078
diagnostic status	0.400	0.400	0.172	0.787	−1.521
task ✻ diagnostic status	0.200	0.200	0.042	0.129	−1.135

Note. Compares models that contain the effect to equivalent models stripped of the effect. Higher-order interactions are excluded. Analysis suggested by Sebastiaan Mathôt.

Model Averaged Q-Q Plot

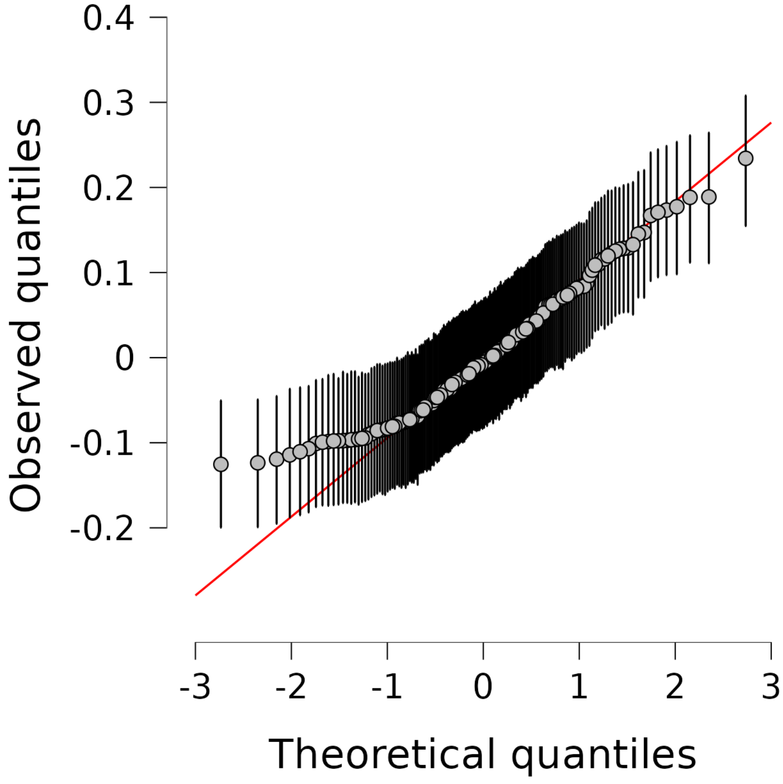

Descriptives

Descriptives

task	diagnostic status	N	Mean	SD	SE	Coefficient of variation
hobbies	ASD	26	0.129	0.100	0.020	0.779
	TD	54	0.114	0.081	0.011	0.711
mealplanning	ASD	26	0.146	0.095	0.019	0.650
	TD	54	0.155	0.102	0.014	0.659

Nonparametric tests

Bayesian Mann-Whitney U Test

	$Log(BF_{10})$	W	Rhat
pit_var	0.888	439.000	1.044
pit_sync	−0.940	609.000	1.023
int_sync	−0.881	609.000	1.009
art_sync	−1.382	666.000	1.013

Note. Result based on data augmentation algorithm with 5 chains of 5000 iterations.

Descriptives

							95% Credible Interval	
	Group	N	Mean	SD	SE	Coefficient of variation	Lower	Upper
pit_var	ASD	26	1035.587	1183.399	232.084	1.143	557.602	1513.572
	TD	54	1982.678	1798.021	244.680	0.907	1491.912	2473.443
pit_sync	ASD	21	0.131	0.055	0.012	0.418	0.106	0.156
	TD	49	0.120	0.083	0.012	0.695	0.096	0.144
int_sync	ASD	21	0.131	0.055	0.012	0.418	0.106	0.156
	TD	49	0.120	0.083	0.012	0.695	0.096	0.144
art_sync	ASD	25	0.139	0.080	0.016	0.573	0.106	0.172
	TD	53	0.135	0.064	0.009	0.473	0.117	0.153

S5: Results comparing dyads

This JASP file contains the assumptions and hypotheses tests on the dyad level. It assesses evidence for and against the following hypotheses:

1. We hypothesise that there will be differences in Silence-to-turn ratio (STR) between ASD-TD dyads compared to TD-TD dyads, such that PTRs will be higher for ASD-TD dyads.
2. We hypothesise that there will be differences in Turn-taking gaps (TTG) between ASD-TD dyads compared to TD-TD dyads, such that TTGs will be longer for ASD-TD dyads.
3. We hypothesise that there will be less synchrony of pitch in ASD-TD dyads compared to TD-TD dyads.
4. We hypothesise that there will be less synchrony of intensity in ASD-TD dyads compared to TD-TD dyads.

ttg: Bayesian Repeated Measures ANOVA

Model Comparison

Models	P(M)	P(M|data)	$\text{Log}(BF_M)$	$\text{Log}(BF_{10})$	error %
Null model (incl. subject and random slopes)	0.200	0.269	0.389	0.000	
dyad type	0.200	0.352	0.775	0.267	1.510
task + dyad type + task ✻ dyad type	0.200	0.221	0.124	−0.200	36.299
task + dyad type	0.200	0.092	−0.906	−1.077	1.971
task	0.200	0.066	−1.260	−1.403	1.566

Note. All models include subject, and random slopes for all repeated measures factors.

Analysis of Effects

Effects	P(incl)	P(excl)	P(incl|data)	P(excl|data)	$\text{Log}(BF_{incl})$
task	0.400	0.400	0.158	0.621	−1.369
dyad type	0.400	0.400	0.444	0.336	0.279
task ✻ dyad type	0.200	0.200	0.221	0.092	0.877

Note. Compares models that contain the effect to equivalent models stripped of the effect. Higher-order interactions are excluded. Analysis suggested by Sebastiaan Mathôt.

Model Averaged Q-Q Plot

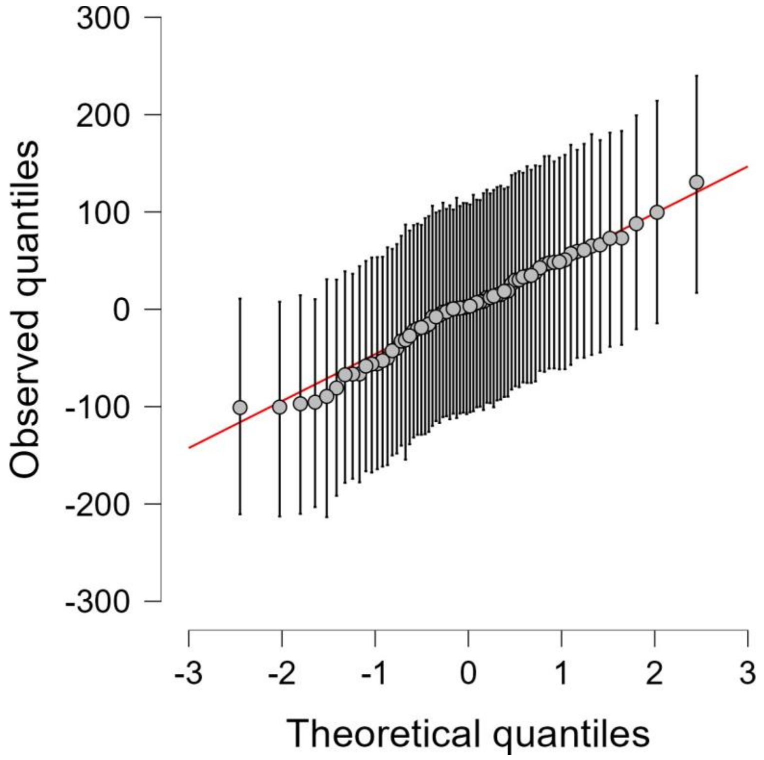

300
200
100
0
-100
-200
-300
Observed quantiles
-3
-2
-1
0
1
2
3
Theoretical quantiles

Descriptives

task	dyad type	N	Mean	SD	SE	Coefficient of Variation	95% Credible Interval	
							Lower	Upper
hobbies	mixed	21	160.000	106.222	23.180	0.664	111.648	208.352
	non-autistic	14	123.429	112.557	30.082	0.912	58.440	188.417
mealplanning	mixed	21	187.048	131.319	28.656	0.702	127.272	246.823
	non-autistic	14	86.857	107.828	28.818	1.241	24.599	149.115

str: Bayesian Repeated Measures ANOVA

Model Comparison

Models	P(M)	P(M|data)	$\text{Log}(\text{BF}_M)$	$\text{Log}(\text{BF}_{10})$	error %
Null model (incl. subject and random slopes)	0.200	0.011	−3.073	0.000	
task + dyad type + task ∗ dyad type	0.200	0.720	2.329	4.141	3.368
task + dyad type	0.200	0.133	−0.490	2.451	12.497
task	0.200	0.125	−0.557	2.394	0.725
dyad type	0.200	0.011	−3.127	−0.054	2.782

Note. All models include subject, and random slopes for all repeated measures factors.

Analysis of Effects

Effects	P(incl)	P(excl)	P(incl|data)	P(excl|data)	$\text{Log}(\text{BF}_{\text{incl}})$
task	0.400	0.400	0.258	0.022	2.449
dyad type	0.400	0.400	0.144	0.137	0.049
task ∗ dyad type	0.200	0.200	0.720	0.133	1.690

Note. Compares models that contain the effect to equivalent models stripped of the effect. Higher-order interactions are excluded. Analysis suggested by Sebastiaan Mathôt.

Model Averaged Q-Q Plot

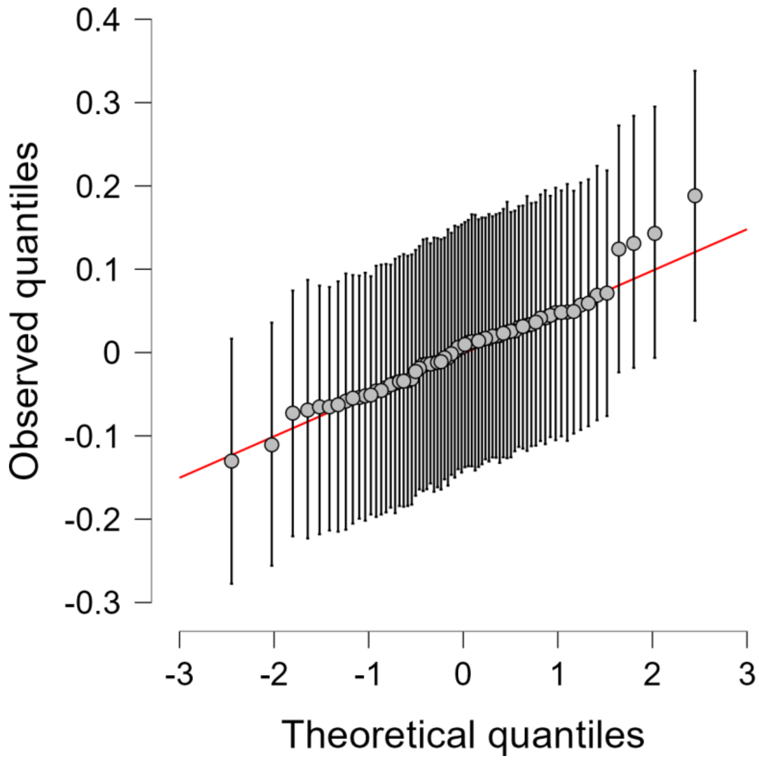

Descriptives

task	dyad type	N	Mean	SD	SE	Coefficient of Variation	95% Credible Interval	
							Lower	Upper
hobbies	mixed	21	0.130	0.158	0.035	1.224	0.057	0.202
	non-autistic	14	0.096	0.145	0.039	1.513	0.012	0.179
mealplanning	mixed	21	0.232	0.219	0.048	0.943	0.132	0.331
	non-autistic	14	0.096	0.132	0.035	1.375	0.020	0.173

pit_sync_MEA: Bayesian Repeated Measures ANOVA

Model Comparison

Models	P(M)	P(M|data)	$Log(BF_M)$	$Log(BF_{10})$	error %
Null model (incl. subject and random slopes)	0.200	0.462	1.235	0.000	
dyad type	0.200	0.320	0.634	−0.367	1.491
task	0.200	0.111	−0.691	−1.424	0.897
task + dyad type	0.200	0.078	−1.079	−1.776	1.906
task + dyad type + task ∗ dyad type	0.200	0.028	−2.171	−2.814	3.366

Note. All models include subject, and random slopes for all repeated measures factors.

Analysis of Effects

Effects	P(incl)	P(excl)	P(incl|data)	P(excl|data)	$Log(BF_{incl})$
task	0.400	0.400	0.190	0.783	−1.418
dyad type	0.400	0.400	0.399	0.574	−0.364
task ∗ dyad type	0.200	0.200	0.028	0.078	−1.039

Note. Compares models that contain the effect to equivalent models stripped of the effect. Higher-order interactions are excluded. Analysis suggested by Sebastiaan Mathôt.

Model Averaged Q-Q Plot

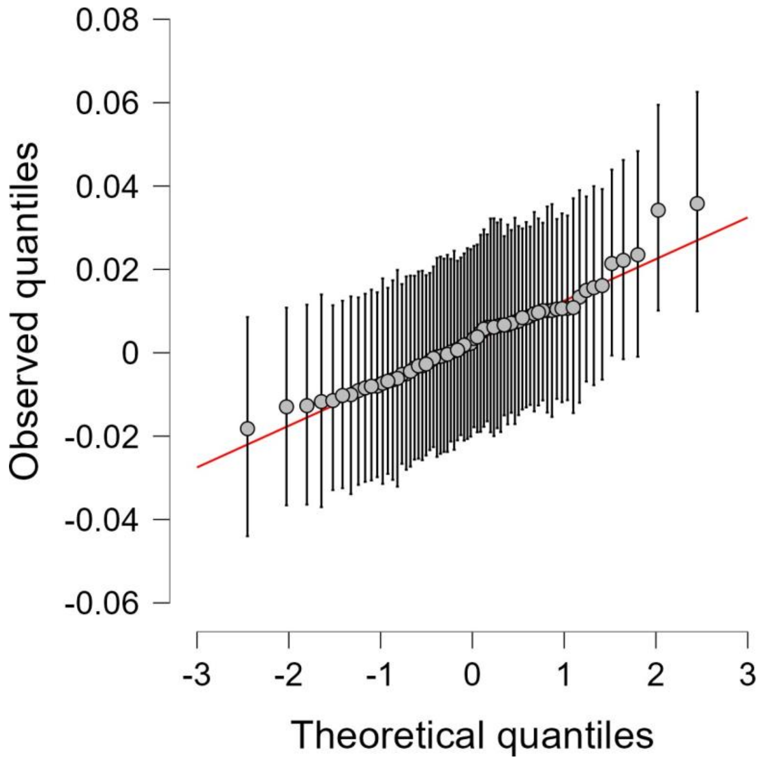

Descriptives

task	dyad type	N	Mean	SD	SE	Coefficient of Variation	95% Credible Interval	
							Lower	Upper
hobbies	mixed	21	0.194	0.022	0.005	0.111	0.185	0.204
	non-autistic	14	0.204	0.026	0.007	0.126	0.189	0.219
mealplanning	mixed	21	0.196	0.019	0.004	0.098	0.187	0.204
	non-autistic	14	0.203	0.021	0.006	0.105	0.190	0.215

int_sync_MEA: Bayesian Repeated Measures ANOVA

Model Comparison

Models	P(M)	P(M|data)	$\text{Log}(\text{BF}_M)$	$\text{Log}(\text{BF}_{10})$	error %
Null model (incl. subject and random slopes)	0.200	4.741×10^{-4}	−6.267	0.000	
task + dyad type	0.200	0.604	1.810	7.150	2.207
task + dyad type + task ∗ dyad type	0.200	0.268	0.381	6.337	4.379
task	0.200	0.125	−0.560	5.574	0.785
dyad type	0.200	0.002	−4.681	1.585	10.261

Note. All models include subject, and random slopes for all repeated measures factors.

Analysis of Effects

Effects	P(incl)	P(excl)	P(incl|data)	P(excl|data)	$\text{Log}(\text{BF}_{incl})$
task	0.400	0.400	0.729	0.003	5.567
dyad type	0.400	0.400	0.607	0.125	1.576
task ∗ dyad type	0.200	0.200	0.268	0.604	−0.813

Note. Compares models that contain the effect to equivalent models stripped of the effect. Higher-order interactions are excluded. Analysis suggested by Sebastiaan Mathôt.

Model Averaged Q-Q Plot

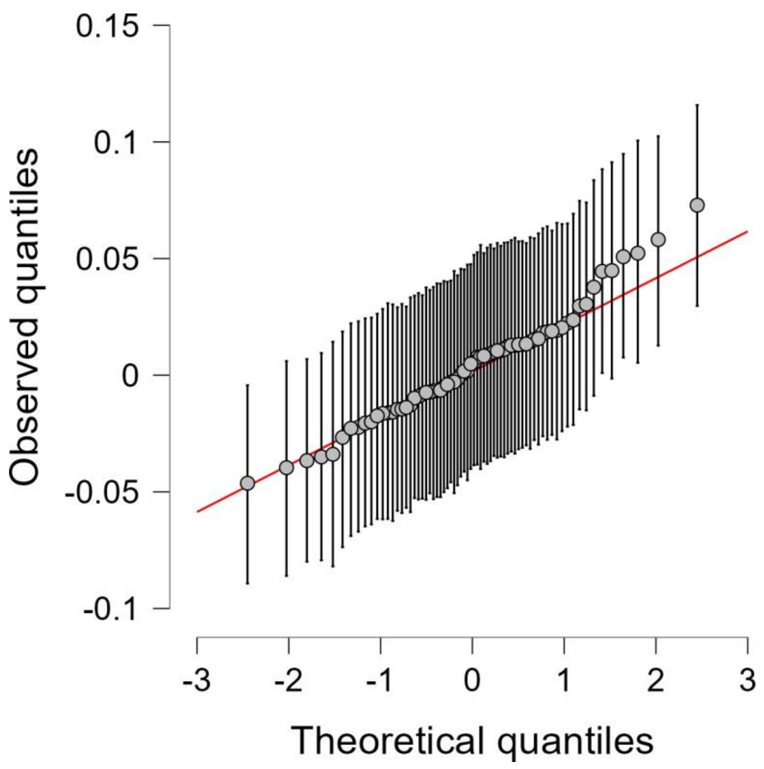

Descriptives

task	dyad type	N	Mean	SD	SE	Coefficient of Variation	95% Credible Interval	
							Lower	Upper
hobbies	mixed	21	0.401	0.054	0.012	0.134	0.377	0.426
	non-autistic	14	0.361	0.034	0.009	0.095	0.342	0.381
mealplanning	mixed	21	0.366	0.041	0.009	0.111	0.348	0.384
	non-autistic	14	0.337	0.032	0.009	0.095	0.319	0.356

S6: Results comparing dyads excluding male dyads

Bayesian Contingency Tables

Contingency Tables

		gen_com		
dyad type		female	mixed	Total
mixed	Count	4.000	13.000	17.000
	% within row	23.529 %	76.471 %	100.000 %
non-autistic	Count	7.000	7.000	14.000
	% within row	50.000 %	50.000 %	100.000 %
Total	Count	11.000	20.000	31.000
	% within row	35.484 %	64.516 %	100.000 %

Bayesian Contingency Tables Tests

	Value
Log (BF_{10}) Independent multinomial	0.206
N	31

Note. For all tests, the alternative hypothesis specifies that group *mixed* is not equal to *non-autistic*
.

ttg: Bayesian Repeated Measures ANOVA

Model Comparison

Models	P(M)	P(M|data)	Log(BF) M	Log(BF 10)	error %
Null model (incl. subject and random slopes)	0.200	0.275	0.418	0.000	
dyad type	0.200	0.405	1.001	0.386	2.222
task + dyad type + task ✻ dyad type	0.200	0.132	-0.494	-0.732	3.645
task + dyad type	0.200	0.111	-0.693	-0.906	3.388
task	0.200	0.077	-1.104	-1.280	1.817

Note. All models include subject, and random slopes for all repeated measures factors.

Analysis of Effects

Effects	P(incl)	P(excl)	P(incl|data)	P(excl|data)	Log(BF) incl
task	0.400	0.400	0.188	0.680	-1.288
dyad type	0.400	0.400	0.516	0.352	0.384
task ✻ dyad type	0.200	0.200	0.132	0.111	0.174

Note. Compares models that contain the effect to equivalent models stripped of the effect. Higher-order interactions are excluded. Analysis suggested by Sebastiaan Mathôt.

Model Averaged Q-Q Plot

Descriptives

Descriptives

task	dyad type	N	Mean	SD	SE	Coefficient of Variation	95% Credible Interval Lower	Upper
hobbies	mixed	14	161.714	98.039	26.202	0.606	105.108	218.320
	non-autistic	14	123.429	112.557	30.082	0.912	58.440	188.417
mealplanning	mixed	14	188.000	103.395	27.633	0.550	128.302	247.698
	non-autistic	14	86.857	107.828	28.818	1.241	24.599	149.115

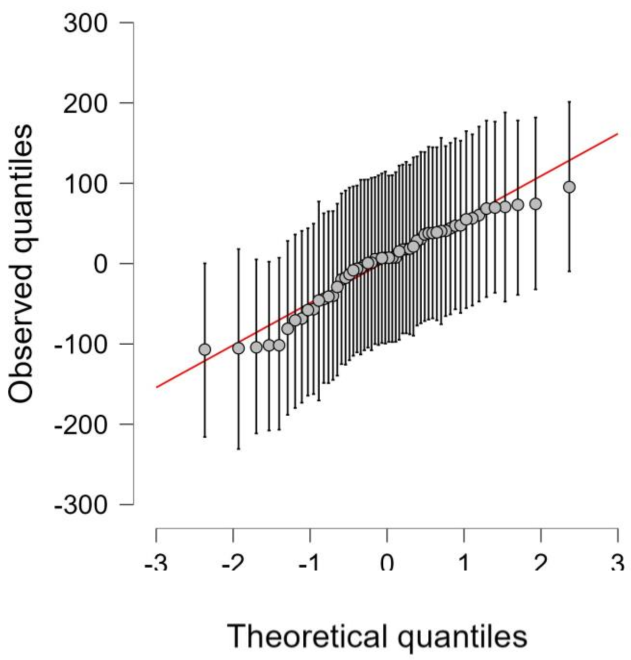

str: Bayesian Repeated Measures ANOVA

Model Comparison

Models	P(M)	P(M|data)	Log(BF) M	Log(BF 10)	error %
Null model (incl. subject and random slopes)	0.200	0.039	-1.805	0.000	
task + dyad type + task ✻ dyad type	0.200	0.656	2.033	2.810	4.135
task + dyad type	0.200	0.142	-0.415	1.277	1.723
task	0.200	0.115	-0.657	1.066	0.799
dyad type	0.200	0.048	-1.603	0.193	1.436

Note. All models include subject, and random slopes for all repeated measures factors.

Analysis of Effects

Effects	P(incl)	P(excl)	P(incl|data)	P(excl|data)	Log(BF) incl
task	0.400	0.400	0.256	0.087	1.076
dyad type	0.400	0.400	0.190	0.154	0.206
task ✻ dyad type	0.200	0.200	0.656	0.142	1.533

Note. Compares models that contain the effect to equivalent models stripped of the effect. Higher-order interactions are excluded. Analysis suggested by Sebastiaan Mathôt.

Model Averaged Q-Q Plot

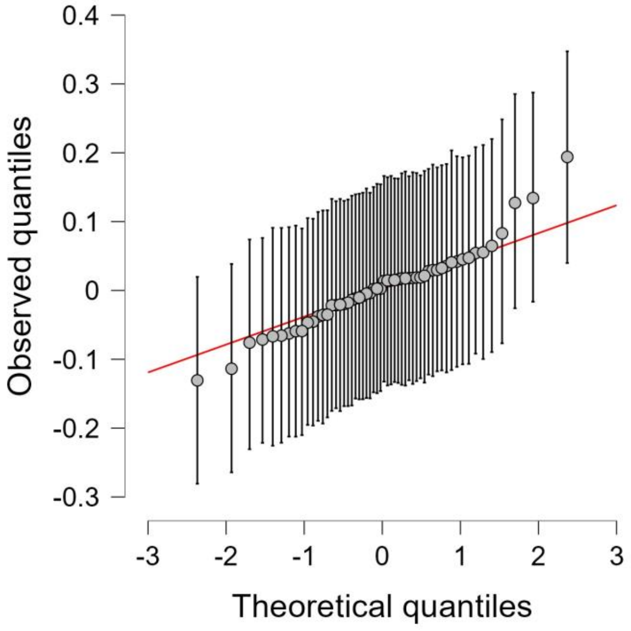

Descriptives

Descriptives

task	dyad type	N	Mean	SD	SE	Coefficient of Variation	95% Credible Interval Lower	95% Credible Interval Upper
hobbies	mixed	14	0.138	0.142	0.038	1.030	0.056	0.220
	non-autistic	14	0.096	0.145	0.039	1.513	0.012	0.179
mealplanning	mixed	14	0.248	0.201	0.054	0.809	0.132	0.364
	non-autistic	14	0.096	0.132	0.035	1.375	0.020	0.173

pit_sync_MEA: Bayesian Repeated Measures ANOVA

Model Comparison

Models	P(M)	P(M|data)	Log(BF M)	Log(BF 10)	error %
Null model (incl. subject and random slopes)	0.200	0.523	1.479	0.000	
dyad type	0.200	0.240	0.233	-0.780	1.389
task	0.200	0.147	-0.373	-1.270	1.573
task + dyad type	0.200	0.067	-1.248	-2.056	2.717
task + dyad type + task ✻ dyad type	0.200	0.023	-2.361	-3.123	2.966

Note. All models include subject, and random slopes for all repeated measures factors.

Analysis of Effects

Effects	P(incl)	P(excl)	P(incl|data)	P(excl|data)	Log(BF incl)
task	0.400	0.400	0.214	0.763	-1.272
dyad type	0.400	0.400	0.307	0.670	-0.781
task ✻ dyad type	0.200	0.200	0.023	0.067	-1.067

Note. Compares models that contain the effect to equivalent models stripped of the effect. Higher-order interactions are excluded. Analysis suggested by Sebastiaan Mathôt.

Model Averaged Q-Q Plot

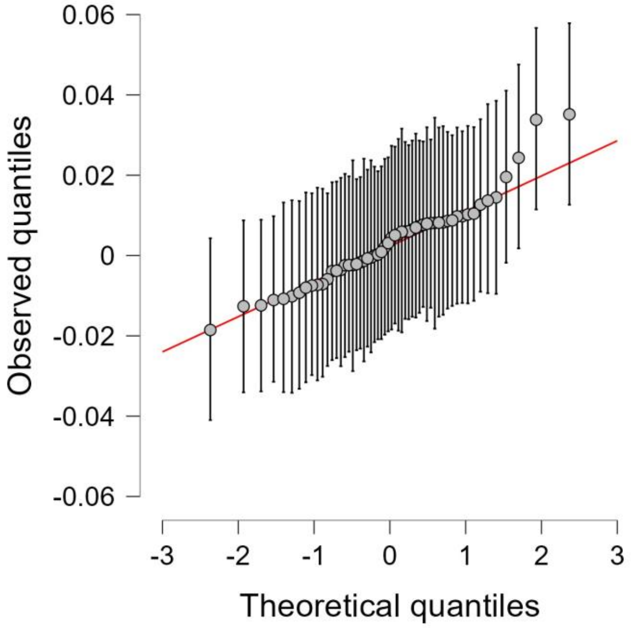

Descriptives

Descriptives

task	dyad type	N	Mean	SD	SE	Coefficient of Variation	95% Credible Interval Lower	95% Credible Interval Upper
hobbies	mixed	14	0.202	0.019	0.005	0.092	0.191	0.213
	non-autistic	14	0.204	0.026	0.007	0.126	0.189	0.219
mealplanning	mixed	14	0.201	0.018	0.005	0.089	0.191	0.211
	non-autistic	14	0.203	0.021	0.006	0.105	0.190	0.215

int_sync_MEA: Bayesian Repeated Measures ANOVA

Model Comparison

Models	P(M)	P(M|data)	Log(BF) M	Log(BF 10)	error %
Null model (incl. subject and random slopes)	0.200	0.003	-4.308	0.000	
task + dyad type	0.200	0.612	1.841	5.207	1.132
task + dyad type + task ✻ dyad type	0.200	0.229	0.174	4.226	3.886
task	0.200	0.142	-0.410	3.748	1.022

*Note.*dyad type All models include subject, and random slopes for all repeated measures factors.0.200 0.013 -2.924 1.375 1.688

Model Comparison

Note. All models include subject, and random slopes for all repeated measures factors.

Models	P(M)P(M|data)Log(BF M)	Log(BF 10)	error %

Analysis of Effects

Effects	P(incl)	P(excl)	P(incl|data)	P(excl|data)	Log(BF) incl
task	0.400	0.400	0.754	0.017	3.816
dyad type	0.400	0.400	0.625	0.146	1.457
task ✻ dyad type	0.200	0.200	0.229	0.612	-0.981

Note. Compares models that contain the effect to equivalent models stripped of the effect. Higher-order interactions are excluded. Analysis suggested by Sebastiaan Mathôt.

Model Averaged Q-Q Plot

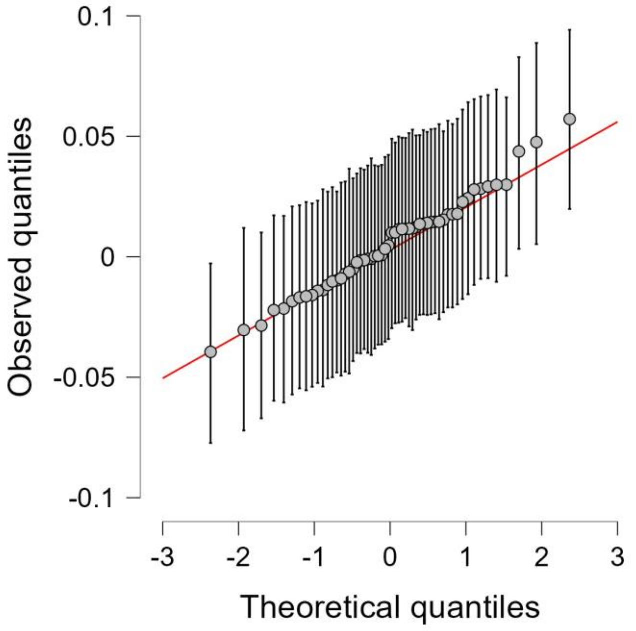

Descriptives

Descriptives

task	dyad type	N	Mean	SD	SE	Coefficient of Variation	95% Credible Interval Lower	95% Credible Interval Upper
hobbies	mixed	14	0.391	0.040	0.011	0.103	0.368	0.415
	non-autistic	14	0.361	0.034	0.009	0.095	0.342	0.381
mealplanning	mixed	14	0.367	0.029	0.008	0.080	0.350	0.384
	non-autistic	14	0.337	0.032	0.009	0.095	0.319	0.356
